# Molecular Engineering of Photosensitizers for Solid‐State Dye‐Sensitized Solar Cells: Recent Developments and Perspectives

**DOI:** 10.1002/open.202300170

**Published:** 2023-10-24

**Authors:** Bommaramoni Yadagiri, Ashok Kumar Kaliamurthy, Kicheon Yoo, Hyeong Cheol Kang, Junyeong Ryu, Francis Kwaku Asiam, Jae‐Joon Lee

**Affiliations:** ^1^ Research Center for Photoenergy Harvesting and Conversion Technology (phct) Department of Energy Materials and Engineering Dongguk University Seoul 04620 Republic of Korea

**Keywords:** Indoor Dye-sensitized photovoltaic, Metal-free organic dye, Ruthenium polypyridyl complex, Solid-state dye-sensitized solar cell, Zinc-porphyrin dye

## Abstract

Dye‐sensitized solar cells (DSSCs) are a feasible alternative to traditional silicon‐based solar cells because of their low cost, eco‐friendliness, flexibility, and acceptable device efficiency. In recent years, solid‐state DSSCs (ss‐DSSCs) have garnered much interest as they can overcome the leakage and evaporation issues of liquid electrolyte systems. However, the poor morphology of solid electrolytes and their interface with photoanodes can minimize the device performance. The photosensitizer/dye is a critical component of ss‐DSSCs and plays a vital role in the device‘s overall performance. In this review, we summarize recent developments and performance of photosensitizers, including mono‐ and co‐sensitization of ruthenium, porphyrin, and metal‐free organic dyes under 1 sun and ambient/artificial light conditions. We also discuss the various requirements that efficient photosensitizers should satisfy and provide an overview of their historical development over the years.

## Graphical abstract

Recent advances in different types of photosensitizers such as ruthenium, porphyrin, and organic sensitizers are reviewed. The design strategies to develop highly efficient sensitizers and their photovoltaic performance with co‐sensitization under 1‐sun and artificial light conditions are discussed.

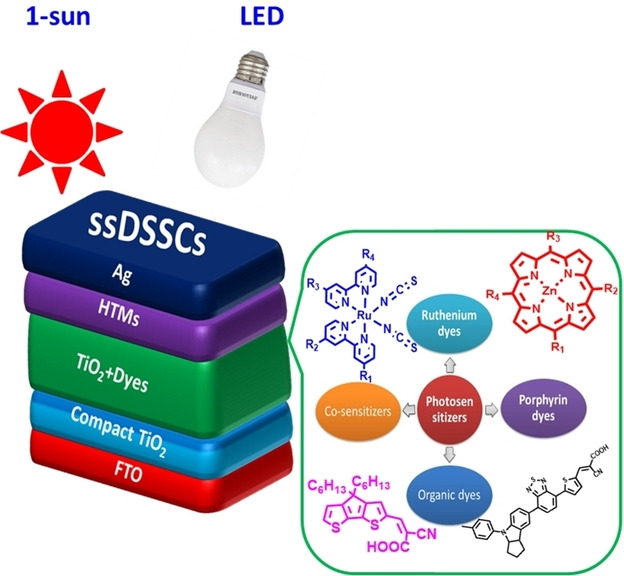

## 1 Introduction

**Figure 1:**
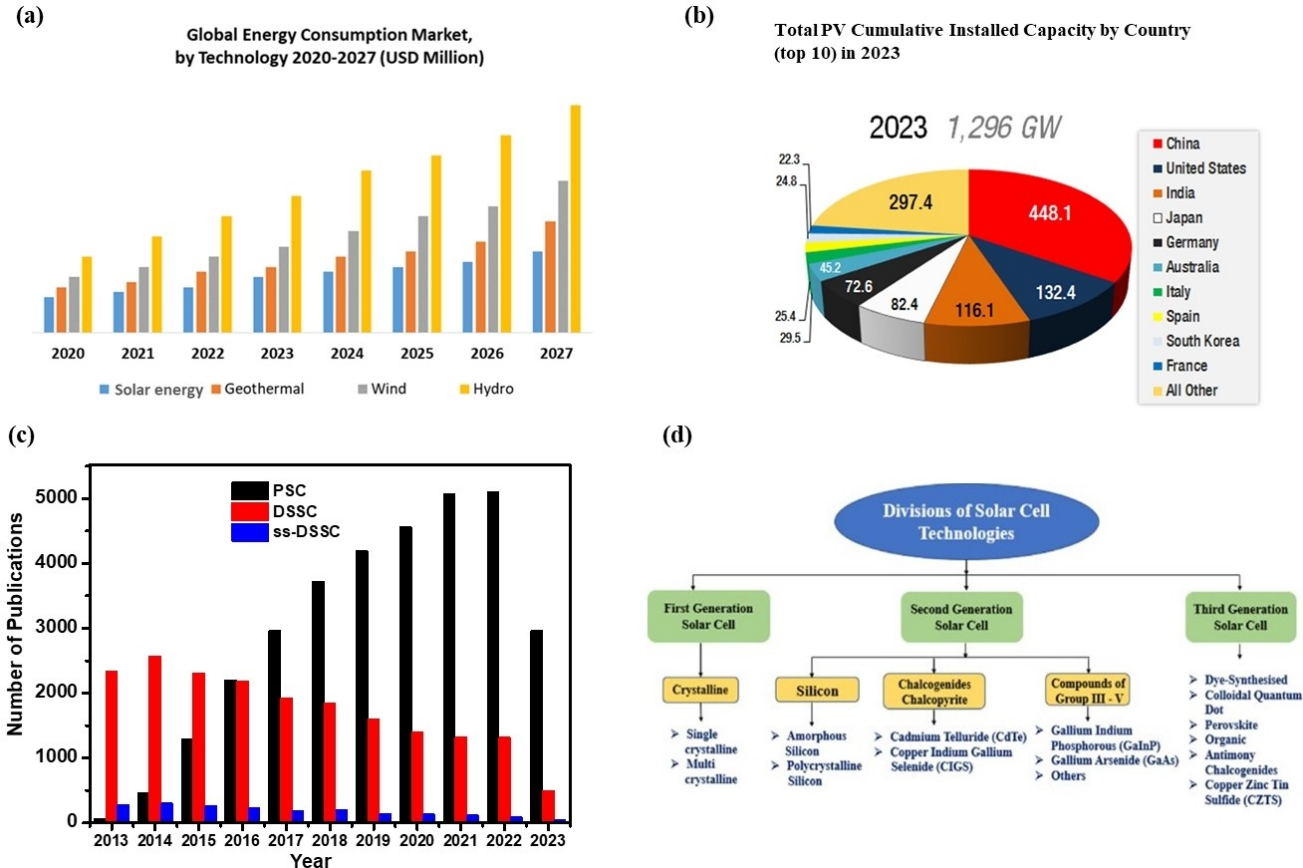
(a) Global energy consumption market with respect to various available energy sources and (b) Top ten countries installed PV technology by 2023. Reproduced from Ref. [7] Copyright (2023), with permission from National Renewable Energy Laboratory. (c) The number of research articles published for year in the fields of PSC, DSSC and ss‐DSSC. Source: Clarivate‐web of science. (d) Classification of solar cell technologies. Reproduced from Ref. [42] Copyright (2022), with permission from Elsevier.

Increasing energy demands and the finite nature of fossil fuels is adversely affecting the global environment. Solar energy has been recognized as the most eco‐friendly, renewable, and readily available energy source compared to other available energy sources on the earth (Figure 1a).[^1^, ^2^] Hence, conversion of solar energy into electrical energy has been proposed as a feasible solution to meet the global energy demands.[^3^, ^4^] The sun releases energy at a rate of 3.8×10^26^ W. Out of this energy, the earth accepts 1.74×10^17^ W (174000 terawatts) of incoming solar radiation in its upper atmosphere. Approximately, 1.08×10^17^ W touches the earth surface, and the remaining amount is reflected into space. Therefore, the solar energy reflected back into the space by the earth surface within 90 min is sufficient to surpass the total global annual primary energy consumption per year.^[5]^

Solar cells, (also known as photovoltaic cells) are solid‐state electrical devices that convert sunlight energy into electrical energy. To achieve high‐efficiency photovoltaic devices, solar cell technologies are being continuously developed by both research communities and industries.^[6]^ Figure 1b shows the total photovoltaic cumulative installed capacity by top 10 countries in 2023.^[7]^ While solar cells have many benefits, they also have some disadvantages including costs and the use of hazardous substrates. Minimum sustainable price (MSP) metric was used to determine the cost related to the production and use of solar cells. According to the NREL report, the MSP of silicon solar panels ranges from 0.34 $/Watt peak (Wp) for panels manufactured in China to 0.54 $/Wp for panels manufactured in Germany. Later some reports suggested an MSP of 0.25–0.27 $/Wp for silicon panels at small scale with possible reductions to 0.18 $/Wp for larger scale. The MSP for third generation solar panels ranges from 0.25 to 0.69 $/Wp.[^8^, ^9^]

Therefore, the third‐generation solar cells, also known as excitonic solar cells, have been attracting more attention because of their facile fabrication methods and low production costs. These include dye‐sensitized solar cells (DSSC), quantum dots solar cells (QDSCs), perovskite solar cells (PSCs), and organic photovoltaics (OPVs).[^10^, ^11^, ^12^, ^13^] In recent years, PV technologies (DSSCs, OPVs, PSCs etc.) considered for use in Internet of Things (IoT) applications have been extensively investigated. The evolution of the number of publications for PSC, DSSC and ss‐DSSC was shown in Figure 1c. Among these, PSCs exhibit great potential for high indoor efficiency devices due to the desirable optoelectronics properties such as tunable bandgap (1.2 eV to 3.5 eV), high absorption coefficients (10^5^ cm^−1^), and small exciton binding energy (<100 meV). Nowadays, the record indoor PCE of PSCs has exceeded 40 % (under 1000 lux), which far exceeds other types of PV cells.[^14^, ^15^, ^16^, ^17^] However, the drawbacks such as toxicity of lead (Pb) and poor stability of perovskite active layer limit their commercialization aspects.[^18^, ^19^]

Dye‐sensitized photovoltaic cells (DSPVs) have been shown to be more effective in ambient light conditions. It has multiple elements of interests, DSPVs can be fabricated by using low‐cost methods such as inkjet or screen printing, which is compatible with conventional roll‐to‐roll techniques. The DSPV modules are semitransparent with a range of different colors. This property, along with their flexibility, makes them extremely attractive for fabricating building integrated photovoltaics (BIPV). Compared to 1‐sun illumination, ambient/indoor lights include light emitting diodes (LEDs), and fluorescence lamps (FLs) exhibit significantly lower intensities with distinct output spectra. Achieving high performance photovoltaics under artificial lighting require fundamentally different device engineering methods.[^20^, ^21^, ^22^] To date, extensive research efforts have been made to enhance the performance of DSPVs under various artificial light illuminations.[^21^, ^23^, ^24^, ^25^]

Michael Grätzel et al. developed a judiciously tailored organic photosensitizer MS5 with the wider spectra response dye XY1b produces a highly efficient and stable DSSC with PCE of 13.5 % under 1‐sun and PCE of 34.5 % under ambient light illuminations.^[26]^ Recently, Y.‐L. Lee et al. developed various co‐sensitized tandem structure DSPVs using D35 + XY1b as top cell and XY1b + Y123 as bottom cell, achieving a high PCE of 36.27 % under FL illuminations.^[27]^ These studies suggest that DSPVs could give enough current to allow the independent operation of small electronic devices such as smart watches, wireless sensor nodes, consumer electronic devices, wearable devices, and smart meters.

In 1991, Grätzel et al. reported DSSC, which used a method similar to natural photosynthesis and had a notable power‐conversion efficiency (PCE) of 7.1 %.^[28]^ The photo‐electrochemical device comprises a wide band‐gap metal‐oxide semiconductor, i. e., mesoporous TiO_2_ (mTiO_2_), with a Ru‐based organic dye. Novel investigations in this field have led to the development of photoactive materials with improved stability, durability and efficiency using fine synthetic methods such as material design approaches and device interfacial engineering.[^29^, ^30^, ^31^] A widely used redox electrolyte for DSSCs is iodide/tri‐iodide (I^−^/I_3_^−^) in an organic liquid electrolyte. However, I^−^/I_3_^−^ has various disadvantages as well, such as extreme corrosivity, volatility, and tendency to react with sealing materials. Therefore, it is unfavorable and adversely impacts the device stability and durability of DSSCs. The development of solid state Dye‐sensitized solar cells (ss‐DSSCs), where the liquid electrolyte is replaced by solid‐state electrolyte have been a topic of interest.[^32^, ^33^, ^34^] In ss‐DSSCs, the photosensitizer selection is considered one of most important steps because the properties of the dyes have a major impact on the light harvesting ability of the photoelectrode, significantly affecting the incident photon‐to‐current conversion efficacy (IPCE) and current density (J_*SC*_).[^35^, ^36^, ^37^, ^38^, ^39^]

Therefore, many research groups have focused on the development of variety of photosensitizers for ss‐DSSC. In this review the recent progress in photosensitizer/dye for ss‐DSSC under standard solar radiation and ambient light conditions was discussed. The effect of molecular design of photosensitizers on their PV performance under outdoor and indoor light illumination is presented. The commercialization aspects of ss‐DSSC under artificial light applications and critical viewpoints on the future PV technology were discussed.

## 2 DSSC Operation Principles

**Figure 2:**
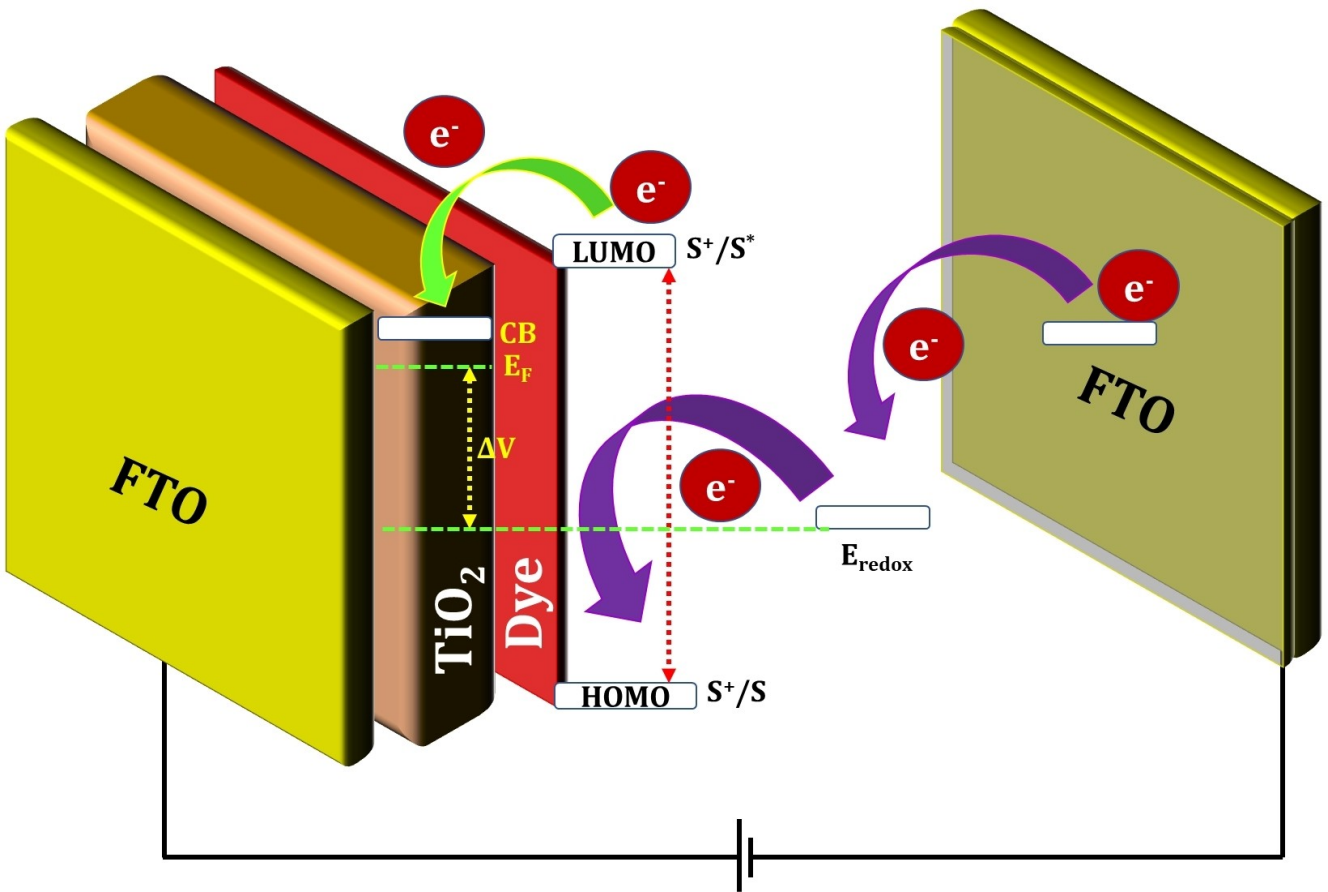
Working principle of a DSSC. S, S^+^, and S* represent the sensitizer in the different states like ground, oxidized, and excited state, respectively; R/R^−^ represents a redox facilitator; CB indicates the conduction band (CB).

Solar cells are classified into three generations such as first‐, second‐, and third‐generation solar cells based on their charge separation, charge collection, and device architecture (Figure 1c).[^40^, ^41^, ^42^] A typical DSSC device consists of five components: (i) a conductive substrate (FTO), (ii) a semiconductor film, (iii) a photosensitizer, (iv) an electrolyte, and (v) a counter electrode (Pt). The efficiency of the DSSC device depends on the performance of each of these components. The working principle of DSSCs is shown in Figure 2. Excitation of the photoactive sensitizer adsorbed on the surface of the semiconductor (PS_as_) [Eq. (1)] helps in injecting excited electrons into the conduction band (CB) of the oxide [Eq. (2)]. Subsequently, the oxidized photoactive sensitizer dye is reduced by the electrolyte, which contains the redox system (R/R^−^) [Eq. (3)]. The injected electron flows *via* the semiconductor system to the back connection and then over the peripheral connection to the counter electrode. The redox species form at the counter electrode [Eq. (4)], which completes the total circuit. Under light illumination with a completed external circuit, the DSSC device establishes a regenerative and stable solar energy‐conversion system. However, it may lead to unwanted transformations, such as, recombination of the injected electrons at the oxidized photosensitizer [Eq. (5)] and TiO_2_ surface [Eq. (6)]. Which can decrease the efficiency.^[31]^

(1) $${PS}_{as}+hv\to {PS}_{as}^{{}^{*}}\;$$

(2) $${PS}_{as}^{{}^{*}}\to {PS}_{as}^{+}\;+e_{injection}^{-}$$

(3) $$PS_{as}^{+}+R^{-}\to {PS}_{as}\;+R\;$$

(4) $$R+\;e_{cathode}^{-}\to R_{cathode}^{-}\;$$

(5) $$e_{injection}^{-}+{PS}_{as}^{+}\;\to PS_{as}\;$$

(6) $$e_{injection}^{-}+R\;\to R_{anode}^{-}\;$$

### 2.1 Charge transfer process in solid‐state DSSCs

**Figure 3:**
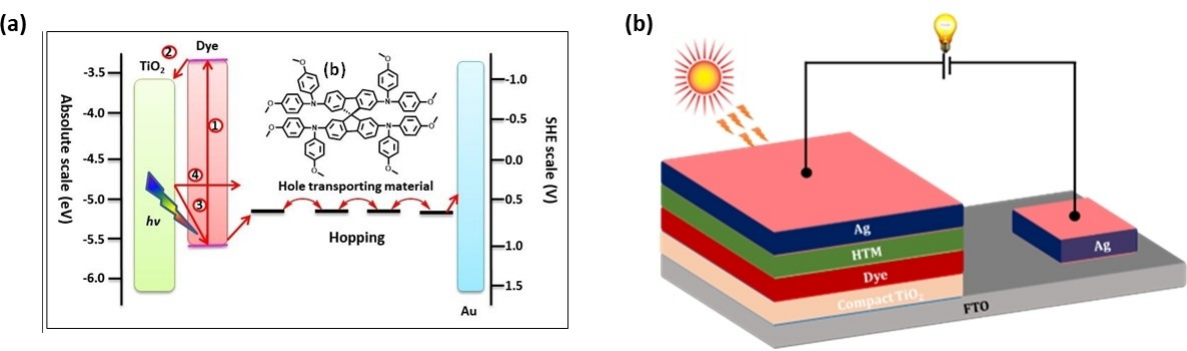
(a) The charge transfer process in ssDSSCs and (b) device architecture of solid‐state DSSC.

Compared to liquid electrolytes, solid‐state electrolytes do not have issues such as leakage, heavy weight, and complex chemistry. Research on ss‐DSSC field has gained much attention in the past decade as they are interesting devices for manufacturing flexible solar cells by applying roll‐to‐roll production. Several research groups have reported a solid‐state organic or p‐type conducting polymers and small molecules as a hole‐transport material (HTMs) for ss‐DSSCs.[^43^, ^44^, ^45^] Figure 3a depicts the charge transfer process in ss‐DSSCs. Which is similar to that of liquid electrolyte DSSCs. However, the charge transfer in ss‐DSSCs continues through the highest occupied molecular orbital (HOMO) of the HTM/solid state electrolyte by a hopping process rather than ionic diffusion method. This is the primary distinction among the ss‐DSSC and liquid state DSSC. The hopping process is a charge transport process; it happens through a disordered landscape of spatial and energetic states. In HTM, the charge transfer process is either an electron or a hole transfer or an ambipolar charge transfer (electrons and holes). The general architecture of the ss‐DSSC device is shown in Figure 3b.^[46]^

## 3 Components of ss‐DSSCs

The architecture of an ss‐DSSC device typically includes various components (Figure 3b), which have been extensively studied, both independently and in combination with other components to improve device performance. This review mostly focuses on recent advances sensitizers for ss‐DSSCs under both 1 sun and ambient light illumination. Additionally, we will provide a brief overview of other components of ss‐DSSC device.

### 3.1 Compact titanium dioxide (Comp‐TiO_2_)

Compact titanium dioxide (Comp‐TiO_2_) plays an important role in the ss‐DSSC devices and forms a blocking interfacial layer among fluorine‐doped tin oxide (FTO) and HTM. This layer prevents the recombination of electrons and holes among the FTO and HTM, respectively, and the rectifying nature of the FTO/compact TiO_2_/HTM junction improves the device performance. Recent, studies have reported the complete performance and merits of compact‐TiO_2_ in ss‐DSSC devices. Several methods have been developed to prepare the comp‐TiO_2_ layer, such as sputtering, chemical vapor deposition, and sol‐gel coating. However, these methods afford substandard blocking interfacial layers compared to those obtained through spray pyrolysis.[^47^, ^48^, ^49^, ^50^]

### 3.2 Mesoporous titanium dioxide (mTiO_2_)

The high performance of ss‐DSSC devices first depends on the nanocrystalline semiconductor film, followed by the sensitizer spectral behavior. Several wide‐band‐gap metal oxides such as TiO_2,_ ^[51]^ ZnO, and SnO_2_ ^[52]^ are used for fabricating ss‐DSSC films, all of which have been widely studied.[^53^, ^54^] To fabricate a device with high performance, it is crucial to have a mesoporous metal oxide film with a large surface area. This helps increase the absorption of solar energy under illumination by using only a monolayer of the adsorbed photosensitizer. Additionally, the monolayer of the dye helps avoid the diffusion of excitons into the photosensitizer or metal‐oxide interface and accelerates the nonradiative decay of the excited state to the ground state. Additionally, the mesoporous film improves the interfacial surface area, leading to high absorbance of the visible light from the adsorbed dye monolayer.[^55^, ^56^, ^57^]

### 3.3 Electrolyte

In conventional DSSCs, the electrolyte plays two key roles: photosensitizer regeneration and transporting holes.^[58]^ Typically, liquid electrolytes are commonly used in DSSC applications.[^59^, ^60^] These electrolytes consist of three elements: a solvent, a redox system (ionic conductor), and multiple additives.[^61^, ^62^] The electrolytes must possess specific characteristics such as a low melting point (−20 °C), a high boiling point (100 °C), high chemical and photochemical stability, high dielectric constant, and low viscosity. Various organic solvents have been used as the electrolyte solution, including alcohols, acetonitrile, organic carbonates, and organic nitriles.^[63]^ One of the commonly used electrolytes for DSSCs is the iodide/triiodide couple (I^−^/I_3_^−^), which exhibits slow recombination kinetics into the CB of TiO_2_. In addition, different types of redox mediators have also been studied.

Cobalt‐based (Co^II^/Co^III^) complexes have shown excellent performance in terms of the Nernst potential, high open‐circuit voltage (V_oc_) and high efficiency among other redox mediators for DSSC.[^64^, ^65^, ^66^] However, DSSC devices made with liquid electrolytes have several disadvantages such as electrolyte leakage, sublimation, and evaporation, which directly affect the device stability. To overcome these disadvantages, a quasi‐solid or solid‐state material is used as the electrolyte for ss‐DSSCs since the last decade. In ss‐DSSC devices, hole transporter such as an inorganic or organic HTM is used instead of a liquid electrolyte. Usually, hole transfer occurs directly between the oxidized photosensitizer and the HTM, after which the holes are transferred to the respective electrode *via* the HTM. However, the performance of ss‐DSSC is lower than that of the conventional DSSCs (based on liquid electrolytes), due to inefficient infiltration of the HTM into the semiconductor pores, which minimizes the TiO_2_ film thickness.

**Figure 4:**
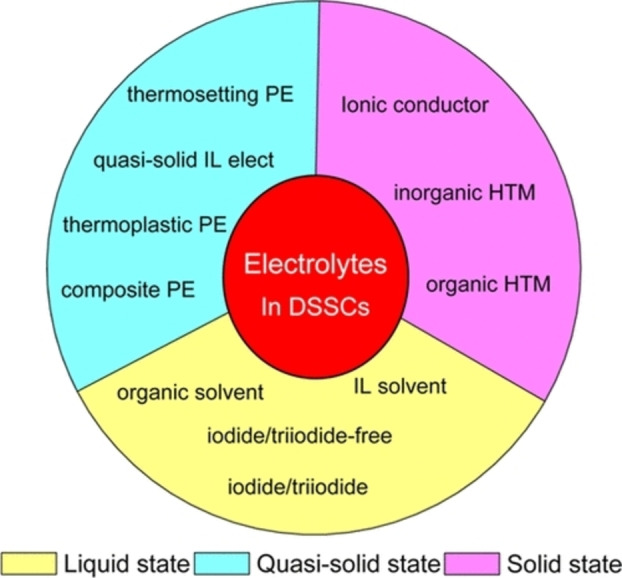
Classification of the electrolytes used in DSSCs. Reproduced from Ref. [58] Copyright (2015), with permission from American Chemical Society.

In addition to HTM, the solid polymer electrolyte (SPE) is also considered as one of the candidates for good electrolytes for all ss‐DSSCs. The most popular methods for solidifying polymer dielectrics are thermosetting and thermoplastic methods. These methods were performed by placing the DSSC device on a hot plate and forming an ss‐DSSC by slow evaporation of the solvent. However, these methods has limitations including this process requires high thermally stable dye and poor electrolyte‐TiO_2_ surface contact, which lead to low PCE.[^67^, ^68^, ^69^, ^70^] To solve the low photoanode thickness problem, “zombie” cells were proposed through the first report by Freitag et. Al, in 2015.^[71]^ Here, a copper phenanthroline (Cu^+/2+^) solid hole conductor was employed to reach a PCE of 8.2 %, higher than that for the liquid electrolyte (6.0 %). Subsequent to this, few works also reported enhancement in PCE with these “zombie” cells, compared to their monolithic counterparts, with the record PCE of 11 % achieved for all‐solid state DSSCs using the same “zombie” architecture.[^46^, ^72^, ^73^, ^74^, ^75^, ^76^] Nonetheless, to increase the efficiency of ss‐DSSCs, it is necessary to use photosensitizers with a very high molar extinction coefficient.[^77^, ^78^, ^79^] Figure 4 illustrates the classification of electrolytes utilized in DSSC.

### 3.4 Photosensitizers

The sensitizer/dye is a crucial component of DSSCs.^[80]^ To improve the performance of the dye, the molecular design of the sensitizer should meet the following conditions: (1) The dye should have anchoring groups such as carboxylate or silyl as an end‐group molecule, which allows strong adsorption and complete coverage of semiconductor oxide surface to prevent electron recombination. (2) The energy level of excited state of the dye molecule should match appropriately with the CB of the TiO_2_ semiconductor for efficient charge injection. (3) The optical absorption of the sensitizer should have a broad and strong absorption profile across the visible to the near‐infrared (NIR) region. High molar extinction coefficients are needed for sufficient light harvesting even with thin electrodes. (4) The highest occupied molecule orbital (HOMO) of the dye should be lower than the redox couple energy levels for efficient regeneration of the dye molecule. (5) The lowest unoccupied molecular orbital (LUMO) of the sensitizer should be higher than the CB of the TiO_2_ semiconductor for efficient electron injection. (6) The sensitizer should have long‐term stability under ambient environmental conditions.[^81^, ^82^]

**Figure 5:**
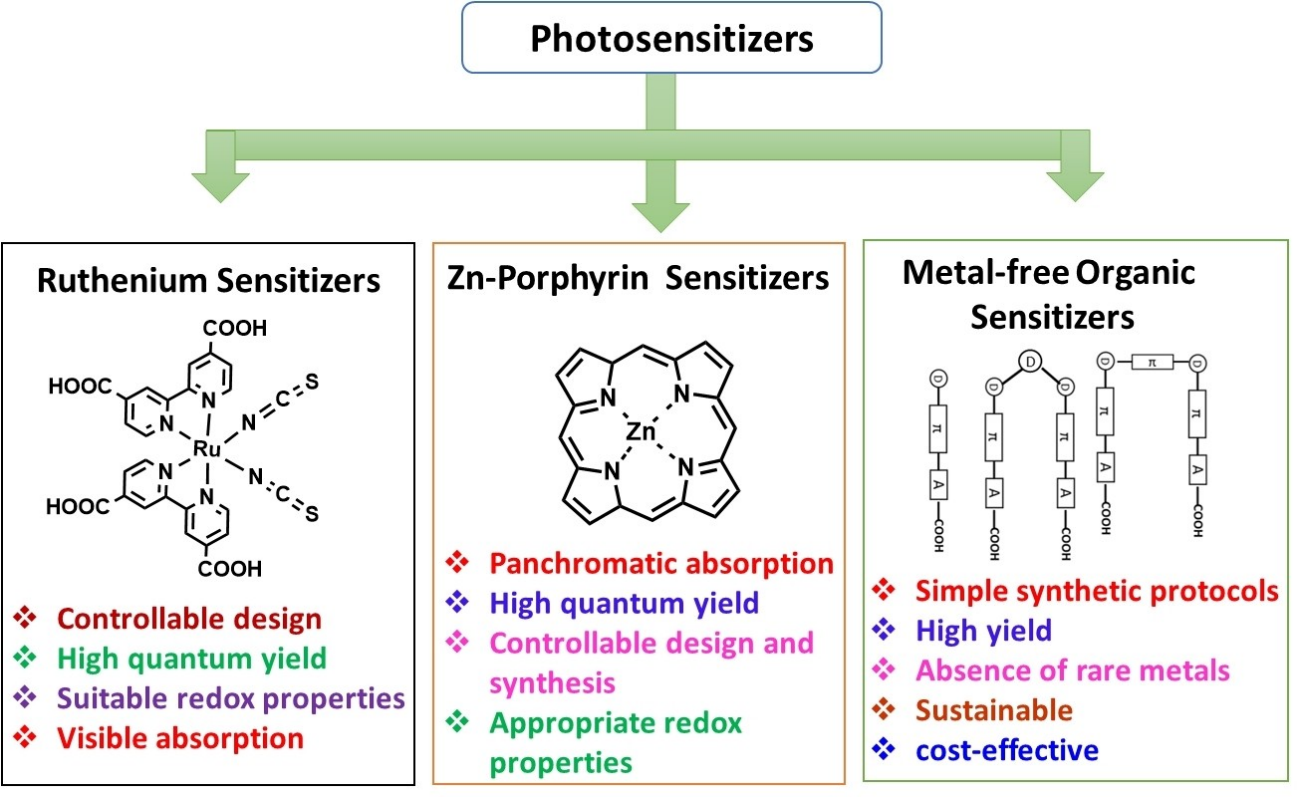
Classification of the photosensitizers in DSSCs.

The donor–spacer–acceptor (D–π–A) molecular design is considered the most efficient for organic sensitizers due to its ability to facilitate the formation of an intramolecular charge transfer (ICT) complex,^[83]^ which facilitates π‐orbital overlap and results in broad and strong visible to NIR absorption. As a result, a large number of studies have focused on increasing the efforts to develop efficient organic sensitizers with the D–π–A structure by changing the donors, spacer units, and acceptor moieties.[^84^, ^85^, ^86^, ^87^, ^88^, ^89^, ^90^, ^91^, ^92^] This review focuses on the recent development made in the field of sensitizers for ss‐DSSC applications. The following three main categories of dyes have been discussed: Ru‐based dyes, porphyrin‐based dyes, and metal‐free organic dyes (Figure 5) with co‐sensitization. The optoelectronic and interfacial properties of their individual components under both 1 sun and ambient light are also briefly discussed.

## 4 Ruthenium‐Based Photosensitizers for ss‐DSSCs

During the early stages of DSSC development, Ruthenium (Ru)‐based organometallic photosensitizers were widely used due to their excellent photovoltaic performance in conventional liquid electrolyte‐based DSSCs.[^93^, ^94^] Because Ru‐based photosensitizers afford a controllable design, synthesis, isolation, conventional purification methods, high quantum yield, suitable photophysical and redox properties, and broad and strong absorption in the visible region, they afford high photovoltaic performance of ss‐DSSCs. The absorption property of Ru‐based photosensitizers completely primarily relies on the metal‐to‐ligand charge transfer (MLCT) process, which allows easy tailoring of most of the broad and strong visible‐light absorption through a suitable modification of ligands.[^95^, ^96^]

In 1991, Grätzel et al. reported the first efficient Ru sensitizer with improved absorption and photovoltaic properties.^[28]^ To increase the efficiency of ss‐DSSCs, the design and development of various novel Ru‐based photosensitizers are being conducted since the last decade. In 1998, Bach et al. reported the use of red dye **1** (Figure 1) for ss‐DSSC applications using amorphous organic HTM 2,2′,7,7′‐tetrakis(*N*, *N*‐di‐p‐methoxyphenyl‐amine)9,9′‐spirobifluorene (spiro‐OMeTAD) as the solid‐state electrolyte. The device achieved a PCE of 0.74 % under illumination of 9.4 mW cm^−2^.^[97]^

Grätzel et al. developed a photosensitizer dye **2** through double protonation of dye **1** and studied its optical, electrochemical, and photovoltaic properties. The ss‐DSSC device based on dye **2** was developed using tert‐butyl‐pyridine (tBP) and lithium ions as additives with spiro‐OMeTAD (HTM) and an improved PCE of 2.56 % was reported.^[98]^ The same group then reported an improved overall device performance of 3.2 % using dye **2** by utilizing silver ions with the sensitizer solution.^[99]^ These results indicate that silver ions can coordinate with the thiocyanate ligands, which can allow the formation of ligand‐bridged dye complexes. The formation of silver complexes results in a more closely packed dye layer and an increased amount of the adsorption of dye units on the TiO_2_ surface, which increases the V_OC_ and short‐circuit current density (J_SC_) of the device and enhances the device efficiency.

Novel amphiphilic dye **3** with hydrophobic spacers was developed by the same group and successfully applied to ss‐DSSCs in 2005 with an improved PCE of 4.0 %.^[100]^ The enhanced performance of devices based on dye **3** can be explained by a compact packing of the dye on the TiO_2_ surface and the hydrophobic isolating chains of the dye that can act as a dense blocking layer between the HTM and TiO_2_. Later, the influence of the hydrocarbon chain length of amphiphilic Ru dyes on the device performance were also studied.^[101]^ The results showed that the longer hydrophobic chain of the sensitizers may increase the distance between TiO_2_ and the HTM, which can minimize the recombination processes. Snaith et al. developed, a novel Ru‐based dye **4**, for ss‐DSSC applications, this dye has a similar structure to that of a well‐known dye **3**.^[102]^ The only difference between dyes **3** and **4** is the replacement of hydrophobic alkyl chains with ion‐coordinating triethylene oxide methyl ether (TEOME) groups, which can suppress the charge recombination and enhance the photovoltaic performance (PCE of 3.8 % for dye **4** as compared to that of 3.2 % for dye **3**).

Further, the photovoltaic performance of dye **4** with lithium ions in ss‐DSSCs has been well investigated.^[103]^ Inhibition of lithium ions on the TiO_2_ surface by the coordination of TEOME groups in dye **4** resulted in an approximately 20 % increase in the device efficiency than that achieved by using dye **3**. However, dye **4** has a TEOME chain as an ion‐coordinating arm on one of the bipyridine ligands, is unstable because of the hydrophilic nature of the ligand that resulted in dye desorption. To overcome this limitation, a novel dye **7**, was prepared by modifying dye **4** and introducing hydrophobic heptyl chains at the end of the alkoxy chains.^[104]^ The ion‐coordinating capability and extra heptyl chains of the TEOME chain also aid in suppressing charge recombination, which improve the device performance (PCE of 4.0 % for dye **7** compared to that of 3.3 % for dye **4**). In 2009, Grätzel et al. reported heteroleptic Ru‐based dye **5** for ss‐DSSCs and studied its photovoltaic performance. Dye **5** consists of a bi‐thiophene group attached to one bipyridine ligand and exhibits a broad visible absorption with the maximum wavelength of 554 nm, a high molar absorption coefficient (ϵ) of 2.42×10^4^ M^−1^ cm^−1^, and suitable electrochemical properties.^[105]^ A PCE of 4.7 % was reported for ss‐DSSCs containing dye **5** and spiro‐OMeTAD HTM under 1‐sun illumination (AM1.5G).

**Figure 6:**
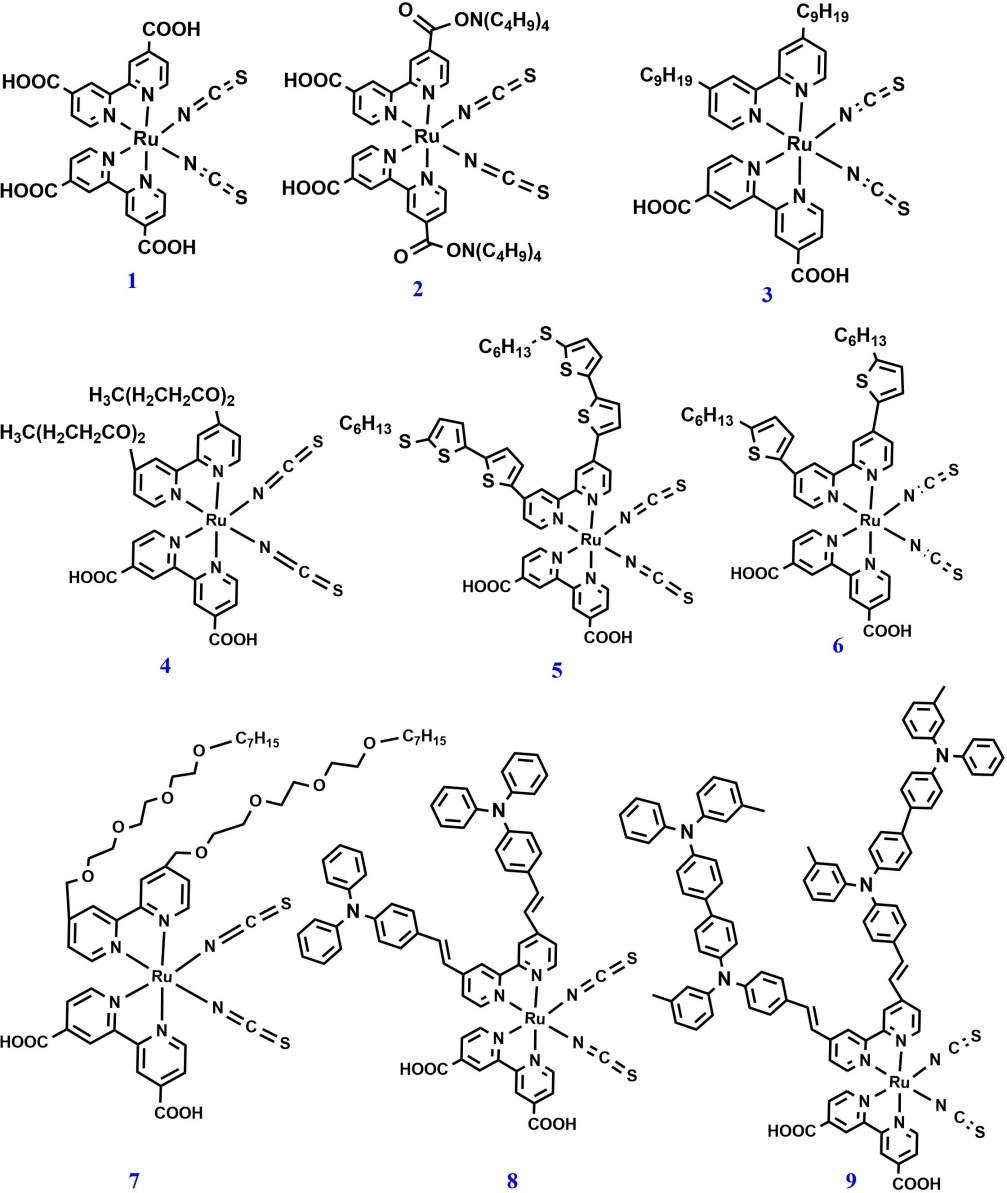
Chemical structures of Ru‐based photosensitizers for ss‐DSSC applications.

Wang *et al*. reported dye **6**, another amphiphilic Ru‐based dye containing alkylthiophene groups for ss‐DSSCs.^[106]^ The structure of dye **6** is similar to that of dye **3** (PCE of 3.2 %), although it has thiophene units on the bipyridine ligand. Dye **6** showed an enhanced efficiency of 4.5 %, which can be attributed to the high molar extinction coefficient of the alkylthiophene groups and increased charge recombination lifetime. In 2007, Thelakkat et al. developed two new dyes **8** and **9**, which are varied by strong electron‐donating antenna groups such as triphenyl amine (TPA) and tetraphenylbenzidine (TPD) for ss‐DSSC applications.^[107]^ Dyes **8** and **9** exhibited remarkable efficiencies of 3.4 % and 1.5 %, respectively, compared to that of standard dye **1** (0.7 %) under the same device conditions. The high efficiency of both dyes is attributed to the TPA and TPD units, which help maintain polarity matching between the nonpolar spiro‐OMeTAD and highly polar Ru‐based dyes. Therefore, improving the interfacial wetting and contact are important for achieving a high performance in ss‐DSSCs. However, the usage of Ru‐based dyes is limited because of their disadvantages such as high cost, less durability owing to the presence of two or three isothiocyanate (NCS) groups, and an absorption maximum of only 550 nm. The chemical structures of some Ru‐based photosensitizers are shown in Figure 6 and their photovoltaic properties are summarized in Table 1.

**Table 1:** Photovoltaic performance of Ru‐based and Zn‐porphyrin‐based sensitizers for ss‐DSSC application.^[a]^

Dye	HTM	Additives	CE	J_SC_ (mA/cm^2^)	V_OC_ (V)	FF	PCE (%)	Reference
Ruthenium‐based dyes								
**1**	spiro‐OMeTAD	Sb, Li	Au	0.32	0.34	0.62	0.74	[97]
**2**	spiro‐OMeTAD	Sb, Li, tBP	Au	5.1	0.91	0.57	2.6	[98]
**2**	spiro‐OMeTAD	Sb, Li, tBP	Au	4.6	0.93	0.71	3.2	[99]
**3**	spiro‐OMeTAD	Sb, Li, tBP	Au	8.3	0.75	0.64	4.0	[100]
**4**	spiro‐OMeTAD	Sb, Li, tBP	Au	6.8	0.88	0.65	3.8	[102]
**7**	spiro‐OMeTAD	Sb, Li, tBP	Au	7.4	0.83	0.65	4.0	[104]
**5**	spiro‐OMeTAD	Li, tBP	Au	9.2	0.83	0.63	4.7	[105]
**6**	spiro‐OMeTAD	Li, tBP	Au	8.2	0.80	0.69	4.5	[106]
**8**	spiro‐OMeTAD	Li, tBP	Au	4.4	0.77	0.34	1.5	[107]
**9**	spiro‐OMeTAD	Li, tBP	Au	9.6	0.76	0.35	3.4	[107]
Zinc Porphyrin‐based dyes								
**10**	spiro‐OMeTAD	‐	Au	5.0	0.73	0.66	2.44	[120]
**11**	spiro‐OMeTAD	‐	Au	5.9	0.79	0.65	3.0	[120]
**12**	spiro‐OMeTAD	Li, tBP, FK102	Au	9.0	0.80	0.64	4.8	[121]
**13**	spiro‐OMeTAD	Li, tBP, FK209	Au	11.0	0.85	0.53	5.1	[122]
**14**	spiro‐OMeTAD	Li, tBP, FK209	Au	10.5	0.79	0.54	4.5	[122]
**15**	[Co(bpy)3]^2+/3+^	‐	Pt	18.1	0.91	0.78	13.0	[123]
**16**	I^−^/I_3_^−^	Li, tBP	Pt	9.4	0.76	0.64	4.6	[30]
**17**	I^−^/I_3_^−^	Li, tBP	Pt	10.6	0.62	0.62	4.1	[30]
**18**	I^−^/I_3_^−^	Li, tBP	Pt	11.7	0.67	0.64	5.0	[30]
**19**	I^−^/I_3_^−^	Li, tBP	Pt	11.2	0.72	0.68	5.2	[30]
**20**	I^−^/I_3_^−^	Li, tBP	Pt	18.6	0.77	0.76	11.0	[124]
**21**	I^−^/I_3_^−^	Li, tBP	Pt	18.4	0.69	0.72	9.3	[125]
**22**	I^−^/I_3_^−^	Li, tBP	Pt	19.1	0.73	0.72	10.1	[125]
**23**	I^−^/I_3_^−^	Li, tBP	Pt	10.05	0.65	0.67	4.38	[126]
**24**	I^−^/I_3_^−^	Li, tBP	Pt	9.94	0.65	0.66	4.27	[126]
**25**	I^−^/I_3_^−^	Li, tBP	Pt	10.81	0.70	0.67	5.13	[126]
**26**	I^−^/I_3_^−^	Li, tBP	Pt	9.42	0.62	0.71	4.2	[127]
**27**	I^−^/I_3_^−^	Li, tBP	Pt	12.21	0.70	0.64	5.5	[127]
**28**	I^−^/I_3_^−^	Li, tBP	Pt	18.95	0.74	0.68	9.61	[128]
**29**	I^−^/I_3_^−^	Li, tBP	Pt	21.13	0.77	0.74	12.1	[129]
**30**	[Co(bpy)3]^2+/3^	‐	Pt	17.34	0.86	0.75	11.4	[130]
**31**	[Co(bpy)3]^2+/3^	‐	Pt	13.69	0.82	0.77	8.74	[130]
**32**	I^−^/I_3_^−^	Li, tBP	Pt	21.43	0.68	0.71	10.51	[131]
**33**	I^−^/I_3_^−^	Li, tBP	Pt	21.25	0.69	0.71	10.45	[131]

[a] HTM: Hole‐transporting material; CE: Counter electrode: J_SC_: Short current density; V_OC_: Open circuit voltage; FF: Fill factor; PCE: Power conversion efficiency.

## 5 Zinc Porphyrin‐Based Photosensitizers for ss‐DSSCs

The second essential metal‐atom‐containing photosensitizers for ss‐DSSC are porphyrin‐based dyes. The optical performance of these dyes can be described by two intense optical absorptions: the Soret band at 400 and 500 nm and intense Q‐bands at 550 and 750 nm. The highly rigid porphyrin core is formed by functionalizing the macrocycle. Usually, dyes with panchromatic absorption are more suitable for ss‐DSSC applications, because molecular engineering of porphyrin dyes allow easy tuning of both bands and afford panchromatic absorption.[^108^, ^109^, ^110^, ^111^, ^112^] In push‐pull porphyrins, the core acts as a π‐spacer unit and is attached between the strong electron‐donor and electron‐acceptor groups. Push‐pull porphyrins allow the expansion of the absorption band as they can harvest a large number of solar photons. The donor moiety “pushes” the electron density toward the porphyrin core, while acceptor moiety on the opposite site “pulls” the electron transferred into TiO_2_. It is believed that this ballistic way strongly promotes electron injection, and hence, they are called “push‐pull” porphyrins.[^113^, ^114^, ^115^, ^116^, ^117^, ^118^, ^119^]

**Figure 7:**
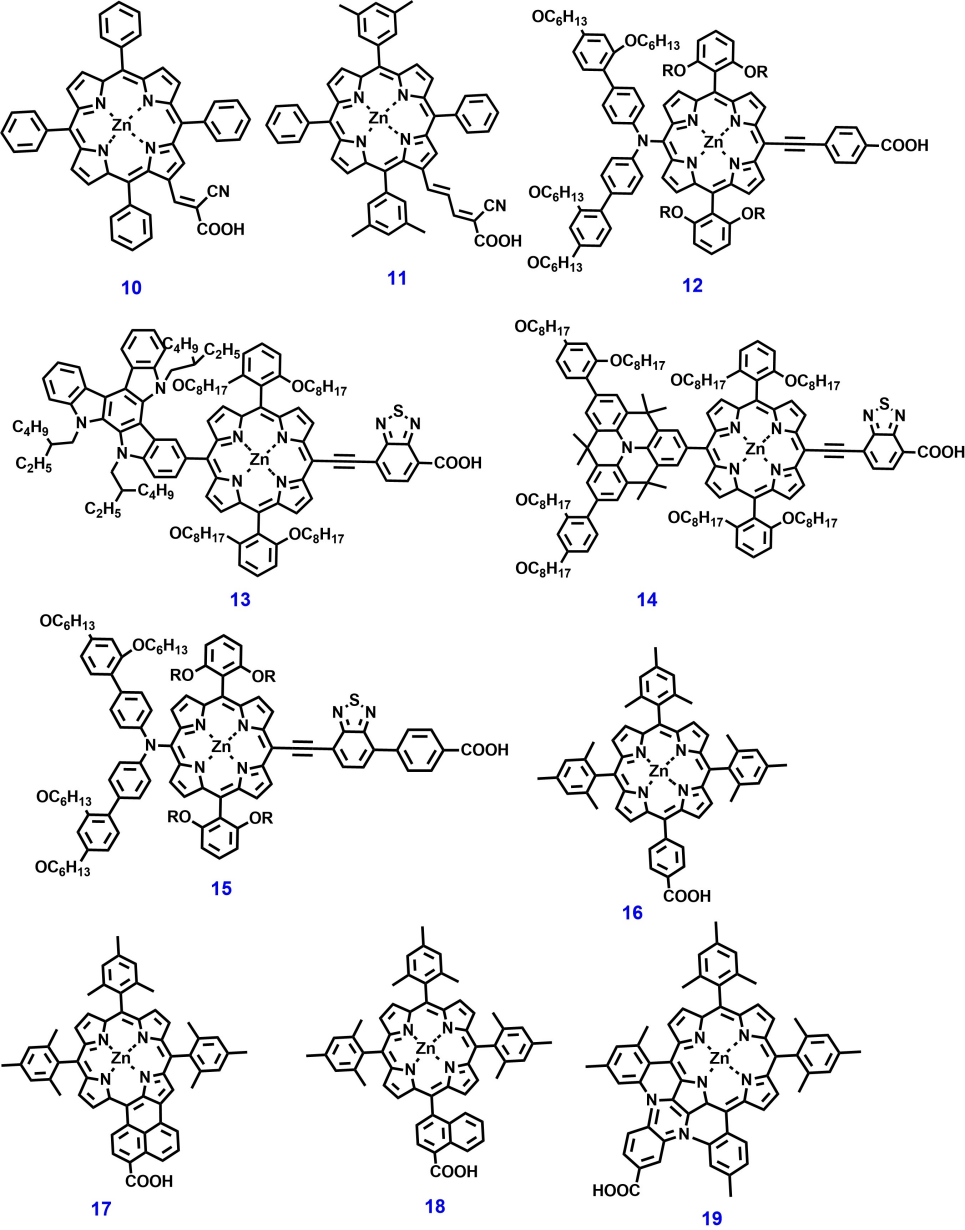
Chemical structures of porphyrin‐based photosensitizers for ss‐DSSC applications.

In 2005, Schmidt‐Mende et al. reported two green porphyrin dyes: cyano‐3‐(2′ (5′,10′,15′,20′‐tetraphenylporphyrinatozinc(II)yl)acrylic acid **10** (Figure 7) and (2‐carboxy‐5‐(2′‐(5′,10′,15′,20′‐tetra(3′′,5′′‐dimethylphenyl)porphyrinatozinc(II))yl)‐penta‐2,4 dienoic acid **11** as sensitizers for ss‐DSSC applications. Both dyes displayed panchromatic absorption and suitable electrochemical properties. ss‐DSSCs with a heterojunction and spiro‐OMeTAD as the HTM afford PCEs of 2.44 % and 3.00 %, respectively.^[120]^ In 2015, Grätzel et al. reported dye **12**, a novel porphyrin‐based push‐pull type for ss‐DSSCs. The solar cell device was made with dye **12** and spiro‐OMeTAD as the HTM reported a PCE of ~4.8 % at AM 1.5G solar irradiation. This push‐pull molecule (dye **12**) was designed by modifying the porphyrin core with appropriate alkoxy chains.^[121]^ An appropriate alkoxy chain at the meso position of dye **12** can influence the electron‐recombination rate, which increases the V_OC_ and PCE of the device.

In 2016, Nazeeruddin et al. reported panchromatic dyes **13** and **14**, which are novel weakly conjugated trans‐A2BC‐type hybrid porphyrin for ss‐DSSC applications.^[122]^ These photosensitizers consist of quinolizino acridine‐ and triazatruxene‐based units, which can be used as the electron‐donating group and as the secondary light‐harvester attached at the meso‐position of the porphyrin central unit, respectively. The ss‐DSSCs made with these molecules as photosensitizers and spiro‐OMeTAD as the HTM afforded PCEs of 4.5 % and 5.1 %, respectively. Dye **13** contains a triazatruxene‐based donor, which exhibits fast electron and hole injection, balanced charge extraction, and less dark current. However, a higher aggregation behavior of the dye lowers the PCE value. Mathew et al. reported a high performance D–π–A structured porphyrin photosensitizer **15**. It demonstrates an exceptional PCE of over 13 % with the cobalt (II/III) redox shuttle under standard AM 1.5G illumination.

Introduction of a benzothiadiazole (BDT)‐functionalized acceptor into the dye structure results in enhanced visible and long wavelength absorption. This enhancement has been analyzed using linear‐response time‐dependent density functional theory (LR‐TDDFT). The significantly improved absorption properties of dye **15** lead to a near‐quantitative light‐harvesting efficiency within the visible‐NIR region (800 nm). As result there is substantial increase in photocurrents in the DSSC.^[123]^

The extension of the π‐conjugation and loss of symmetry in porphyrin core unit leads to an enhancing Q bands absorption peak intensity compared to that of the Soret bands. Based on this strategy, Imahori et al. developed a novel panchromatic photosensitizer **16**–**19**, which shows an improved light absorption in between 400–600 nm. Among the dye compounds used for DSSC, the one constructed with fused five‐membered zinc porphyrin carboxylic acid (dye **17)**, in which the benzene ring act as bridge in the phenyl carboxylic acid was fused to the β‐position of the meso‐tetraphenylporphyrin unit, delivered a PCE of 5.0 %. Similarly, the unsymmetrical π‐conjugated β,β‐edge‐fused porphyrin dye **19** was developed. This dye exhibits a rigid structure and well‐defined molecular length, as well as a relatively broad absorption spectrum. Consequently, the TiO_2_ cell sensitized with dye **19** exhibited a PCE value of 6.3 %.^[30]^

Bessho et al. developed a porphyrin chromophore as π‐bridge into a D–π–A structure dye **20**. It exhibits a unique PCE of 11 % when used as a photosensitizer on a double‐layer TiO_2_ film under AM 1.5G solar irradiation. Additionally, this study also demonstrated that dye **20** shows an improved PV performance, when co‐sensitized on a thin TiO_2_ film with a metal free dye that has a complementary absorption spectral response.^[124]^

**Figure 8:**
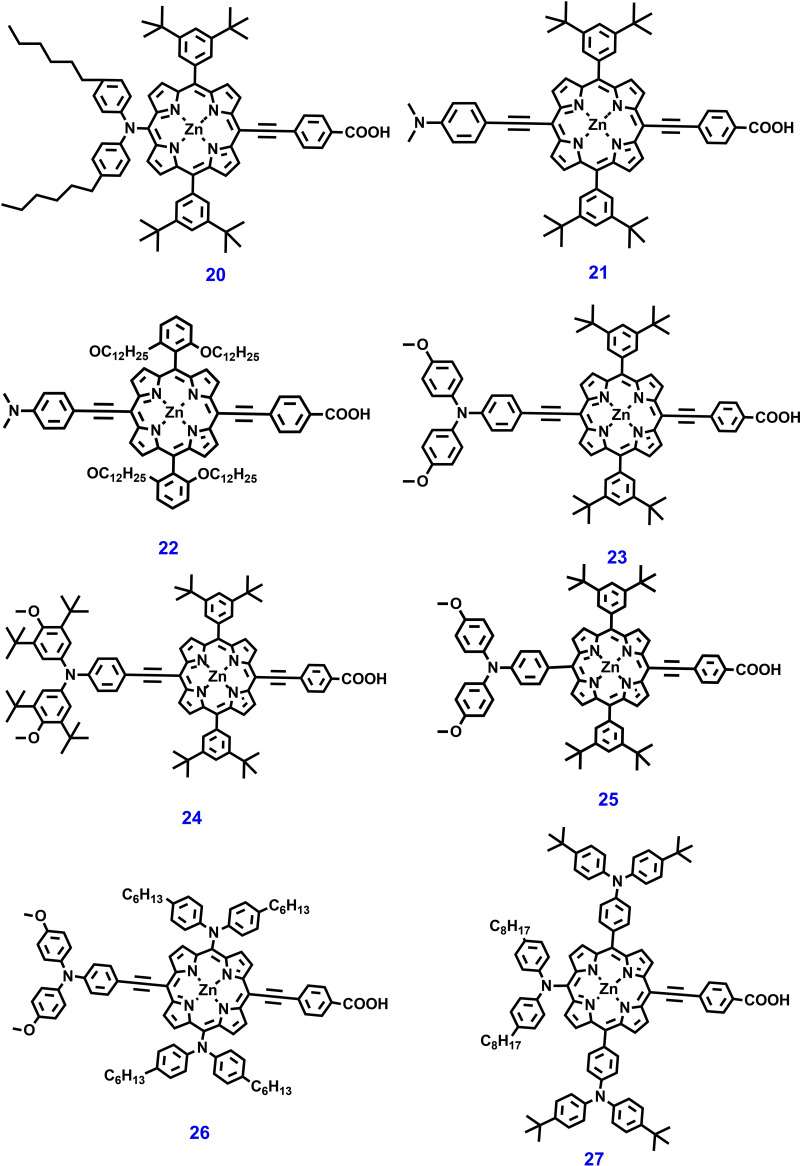
Chemical structures of Zn‐porphyrin‐based photosensitizers for ss‐DSSC applications.

Chang et al. reported a highly efficient porphyrin dyes **21**–**22** (Figure 8), these dyes feature two phenyl groups at meso‐positions of the macrocycle bearing two ortho‐substituted long alkoxyl chains and two meta‐substituted t‐butyl alkyl units respectively for DSSC. The CV study revealed that porphyrin dye **22** has more negative oxidation and reduction potentials than dye **21**. Consequently, the ortho‐substituted porphyrins might have high V_OC_ and fast electron injection compared to their meta‐substituted counterparts. As result, DSSC devices fabricated with dyes **21**–**22** show PCEs of 9.34 % and 10.17 % respectively under simulated AM‐1.5G illumination.^[125]^

Hsieh et al. designed and synthesized a series of porphyrin dyes **23**–**25** with an electron‐donating group (EDG) attached at a meso‐position for DSSCs. The influence of EDG on spectral response, electrochemical and PV properties of these sensitizers was well discussed. Among these, dye **23** and **24** have a triphenylamine conjugated unit at the meso‐position opposite the anchoring group, showing a significant shift in first oxidation towards the negative region implying a reduced HOMO‐LUMO gap. As result, dye **23‐**sensitized TiO_2_‐based DSSC exhibited a PCE of 4.38 %. This study suggests that the direct connection of an alkylated‐diarylamino group into the porphyrin unit produces a substantial enhancement in PV performance.^[126]^

**Figure 9:**
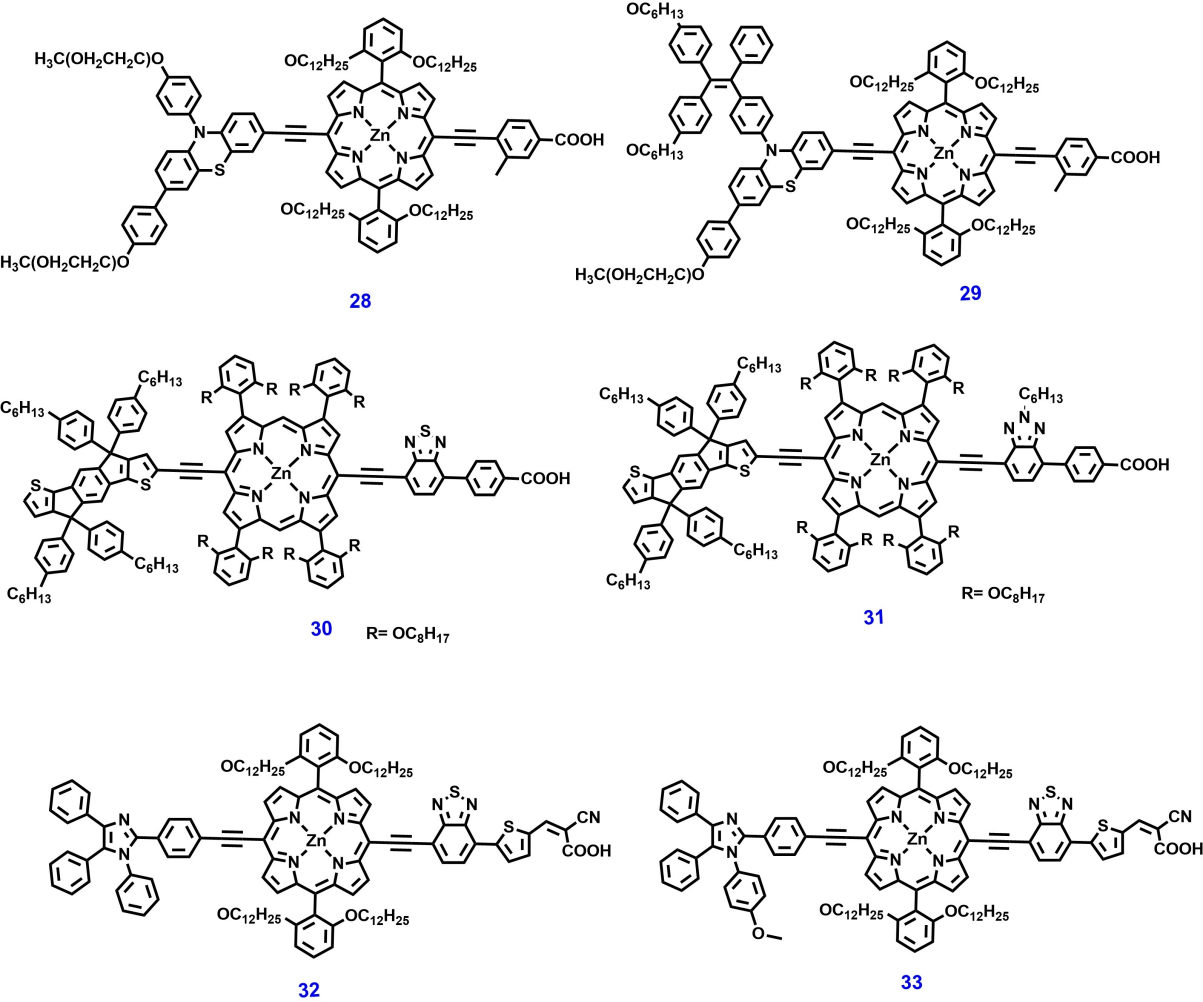
Chemical structures of porphyrin‐based photosensitizers for ss‐DSSC applications.

Wu et al. developed porphyrin dyes **26**–**27** with push‐pull structure for DSSCs. The molecular design of these dyes consists of N, N‐dimethoxy triphenyl amine, para‐hexyl substituted diphenyl amine as a D unit and benzoic acid an acceptor unit. These units are covalently linked to the π‐bridge porphyrin unit. The electrochemical results of dyes **26**–**27** show that the first oxidation occurs at a greater potential than that of the I^−^/I_3_^−^ redox couple and incorporation of EDGs into the porphyrin central core unit facilitates higher electron abstraction. The DSSC device constructed with dye **26** and **27** exhibited a PCEs of 4.2 % and 5.5 % respectively.^[127]^ Lu et al. reported a new porphyrin sensitizer **28** (Figure 9) containing two oligo(ethylene glycol) (TEG) units to the phenothiazine donor. The dye **28** exhibited a high V_OC_ of 752 mV, J_SC_ of 18.53 mA cm^−2^ and PCE of 9.61 % owing to the anti‐aggregation and Li^+^‐trapping capability of the TEG units. Furthermore, a PCE of 10.83 % was achieved by using co‐adsorbent CDCA and metal free organic a co‐sensitizer PT−C6.^[128]^ Recently, the same group developed highly efficient porphyrin dye **29** for DSSC. The dialkoxy‐substituted highly twisted tetraphenylethylene (TPE) attached to donor unit can effectively suppress dye aggregation and charge recombination. As result PV parameters including J_SC_, V_OC_, FF and PCE were significantly improved. Furthermore, a high PCE of 12.15 % has been reported for dye **29** by co‐sensitization and co‐adsorbent method.^[129]^

Recently, double‐fence porphyrin sensitizers with excellent PV properties were reported, in these sensitizers eight alkoxyl chains were incorporated to wrap the porphyrin core and impede the contact of the electrolyte to the TiO_2_. Based on this molecular design, in 2023, Chen et al. developed a double‐fence porphyrin photosensitizers **30** and **31**, featuring dual functional indacenodithiophene (IDT) groups for DSSC. Dye **31** contains benzotriazole (BTA) moiety exhibiting a higher Soret band absorption and slightly blue‐shifted Q band by 8 nm was observed as compared to dye **30**, which contains BTD moiety. As result, the champion device constructed with [Co(bpy)_3_]^3+/2+^ as electrolyte exhibited a PCE of dye **30** (η=11.4 %) than their counter part the dye **31** (η=8.74 %). This study suggests that the IDT donor is effective in enhancing the light collecting efficiency of dyes and suitable aliphatic chain lengths must be carefully chosen to balance molecular aggregation and dye loading.^[130]^

Giribabu et al. designed and synthesized a series of porphyrin sensitizers **32** and **33**, which are distinguished with methoxy group on the phenyl ring of the triphenyl imidazole donor moiety. The optical of these sensitizers were analyzed by the UV visible absorption study, indicating a redshift in the Q‐band and an extension of the absorption onset to 800 nm. Density functional theory (DFT) calculations suggested that the HOMO levels are delocalized in both the triphenyl imidazole donor part and porphyrin core unit, while LUMO is located on auxiliary acceptor BDT unit and anchoring cyanoacetic acid unit. The co‐sensitized DSSC fabricated using dye **33** and a liquid I^−^/I^3−^ redox electrolyte exhibited an outstanding PCE of 10.45 %.^[131]^ The chemical structures of zinc porphyrin‐based dyes are shown in Figures 7 and 9 and their photovoltaic parameters are summarized in Table 1.

## 6 Metal‐Free Organic Dyes for ss‐DSSCs

The third main class of sensitizers for ss‐DSSCs is the metal‐free dye. Compared to Ru‐based dyes, metal‐free organic dyes have several advantages such as simple synthetic protocols, high yield, absence of rare metals, cost‐effective, sustainable, and simple purification methods. The molecular engineering of metal‐free organic dyes can be achieved through some well‐established synthetic methods (Suzuki, Stille, Sonogashira cross coupling etc.).[^132^, ^133^] They afford broad and strong absorption in the visible region and have suitable HOMO and LUMO values, resulting in enhanced photovoltaic properties of dyes. Numerous approaches have been adopted to achieve high‐efficiency DSSC devices, such as by testing various novel dyes.^[134]^

**Figure 10:**
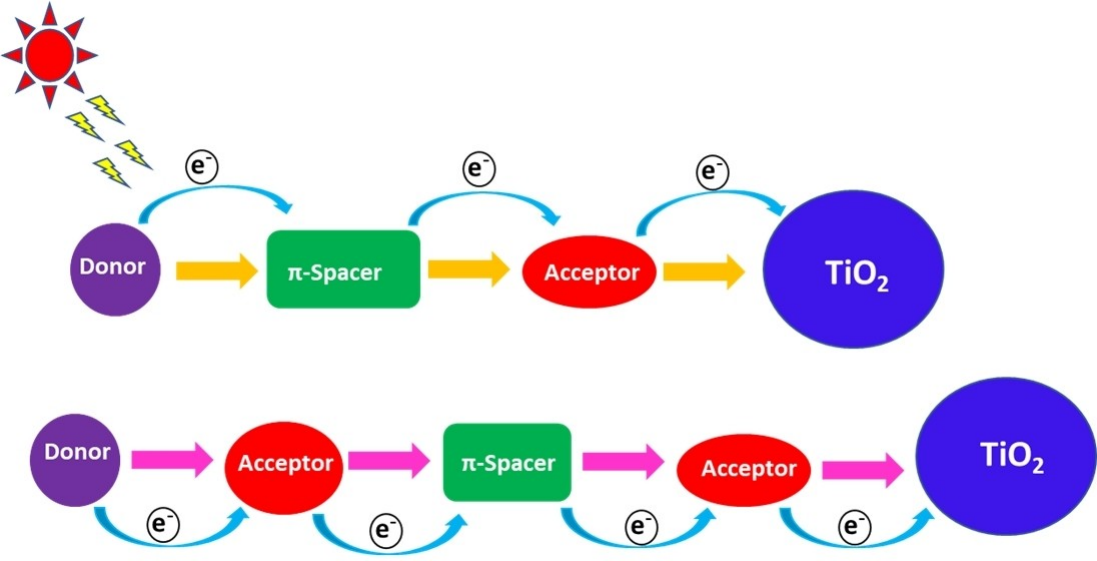
Metal‐free organic dyes with the D–π–A and D–A–π–A molecular architecture.

Metal‐free organic dyes with the D–π–A and D–A–π–A molecular architecture (Figure 10) have been studied for ss‐DSSCs, as they can facilitate the separation of photo‐induced charges.^[135]^ Typically, the donor part is composed of electron‐rich groups, the π‐spacer unit comprises highly rigid conjugated units, and the acceptor unit is composed of electron‐deficient/withdrawing units.^[136]^ The acceptor unit also acts as the anchoring group and can bind to the semiconductor surface. In these molecular architectures, the HOMO of the dye molecule is mainly localized on the donor part, whereas its LUMO is mainly localized on the acceptor part. Upon photoexcitation, electron transfer occurs from the donor to the electron acceptor through the π‐bridge. This process separates the electrons and holes in the dye molecule, which facilitates electron injection into the CB of TiO_2_ and hinders charge recombination between the semiconductor and the oxidized dye.

### 6.1 Organic dyes with cyanoacrylic‐acid anchoring unit:

**Figure 11:**
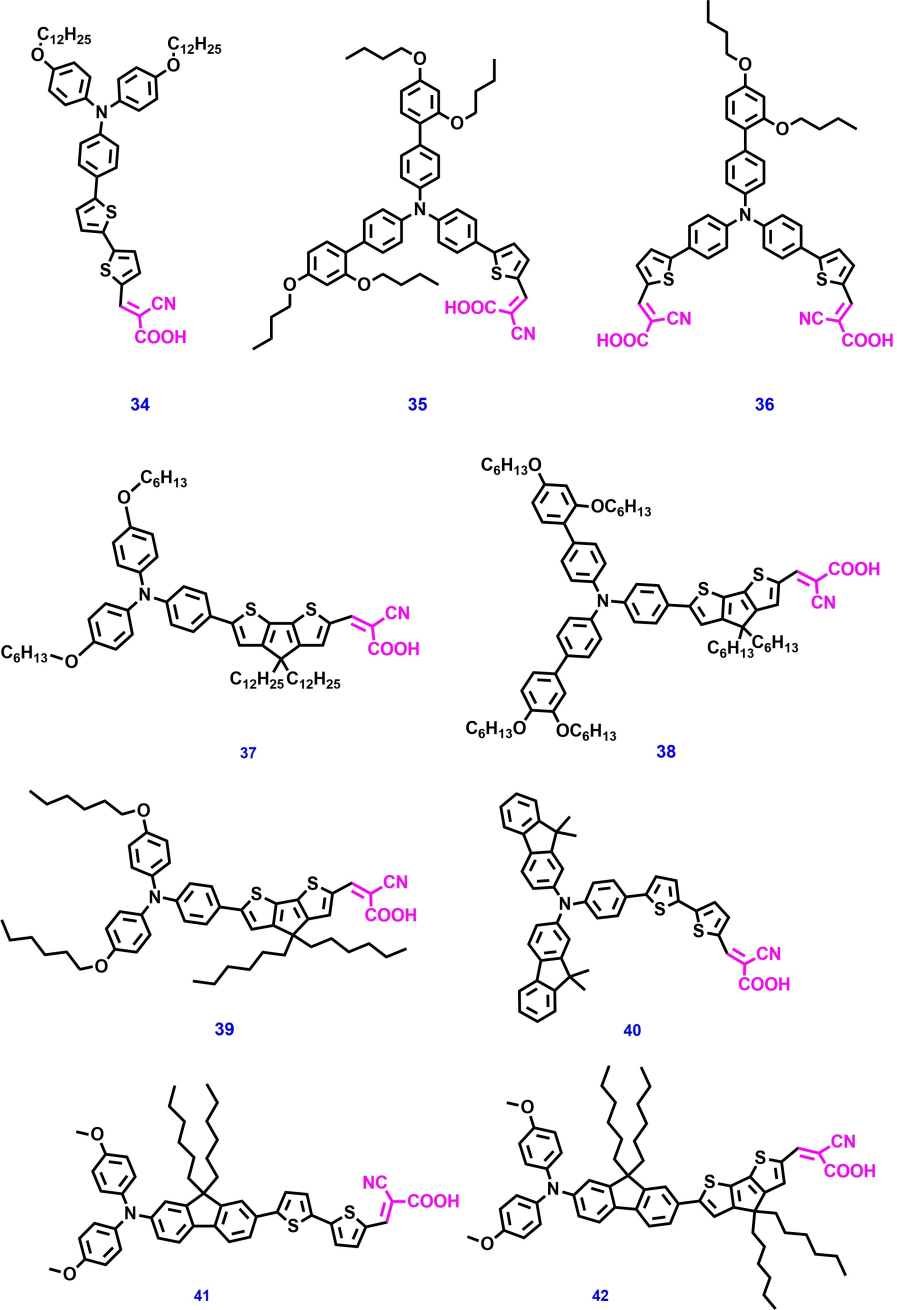
Chemical structures of metal‐free organic photosensitizers for ss‐DSSC applications.

In 2009, Nazeeruddin et al. designed and developed three novel forms of organic dyes **34** (Figure 11) by using different alkyl chains in the triphenylamine unit and used them to investigate the ss‐DSSC performance.^[137]^ The highest PCE of 4.4 % was achieved when spiro‐OMeTAD was used as the HTM molecule in ss‐DSSC devices. Enhanced efficiencies were obtained when alkoxy groups were attached to the donor unit. Because of the interactions with the alkoxy unit, the sensitizer absorbs a large amount of solar energy and affords a high overall conversion efficiency. Again, in 2011, Jiang et al. developed two triphenylamine‐based metal‐free organic sensitizers: **35** with a single anchor group and **36** with two anchor groups for application in ss‐DSSCs.^[138]^ PCEs of 4.5 % for **35** and 4.4 % for **36** were reported using spiro‐OMeTAD as the HTM under standard AM 1.5G illumination.

Additionally, these molecules were studied to investigate their application in liquid‐state DSSCs using iodine‐based electrolyte and high PCEs of 6.74 % and 5.51 %, respectively, were obtained. Even though dye **35** has a lower molar extinction coefficient and a slightly narrower absorption spectrum than those of dye **36**, the performance of the former in ss‐DSSCs is better than that in the latter because of the monolayer molecular structure of dye **35**, which influences the electron lifetime and effectively inhibits electron recombination. Michael Grätzel et al. developed organic dye **37** with D–π–A architecture for ss‐DSSCs and reported a high molar absorption coefficient and a PCE of >6 % using spiro‐OMeTAD as the HTM under AM 1.5G solar irradiation. This dye afforded the highest conversion efficiency (6.08 % by NREL) for heterojunction ss‐DSSC applications. The enhanced performance of dye **37** is owing to the high charge‐collection efficiency over a wide potential range, which was well supported by the IPCE spectra, transient photovoltage, and photocurrent decay measurements.^[139]^

In 2012, De Angelis et al. designed and synthesized a novel triphenyl amine‐based metal free organic dyes **38**, **39**, and **40** for ss‐DSSC applications. This work mostly discusses the interaction between the HTM and the Dye‐sensitized TiO_2_ surface. The novel dyes exhibited strong visible absorption maximum at 471, 481, and 472 nm with high molar absorption coefficients of 45×10^3^, 34×10^3^, and 29×10^3^ M^−1^ cm^−1^, respectively. An ss‐DSSC device was designed using organic D–π–A dyes as photosensitizers and spiro‐OMeTAD as the HTM and a 934 mV open‐circuit potential and PCE of 6.9 % under regular solar conditions (AM1.5G, 100 mW cm^−2^) were obtained.^[140]^ Later, Yiming Cao et al. reported a record PCE of 11 % with stable metal‐free organic dye **38** for ss‐DSSCs under AM1.5G standard conditions. This device comprised a solid HTM composed of a blend of [Cu(tmby)_2_](TFSI)_2_] and [Cu(tmby)_2_](TFSI)]. The amorphous Cu (II/I) conductors conduct holes by prompt hopping infiltrated in a 6.5 μm‐thick mesoscopic TiO_2_ scaffold. This phenomenon is responsible for the high efficiency of dye **38‐**based ss‐DSSCs.

A time‐resolved laser photolysis study revealed the time constants for electron injection from dye **38** into TiO_2_ as well as dye regeneration from transition metal complexes (Cu(I)).^[141]^ In 2013, three novel D–π–A structured organic dyes (**41**, and **42**, Figure 11) were reported by Sellinger et al. The synthesized dyes can separate the TiO_2_ semiconductor from the spiro‐OMeTAD, causing a slow recombination between them, and increase the device performance. Additionally, the quantity and position of alkyl chains on the photosensitizer plays a crucial role in avoiding recombination. ss‐DSSC devices made using dyes **41**, **42**, and **43** as photosensitizers and spiro‐OMeTAD as the HTM afforded PCEs of 4.9 %, 5.9 %, and 6.3 %, respectively.^[142]^

**Figure 12:**
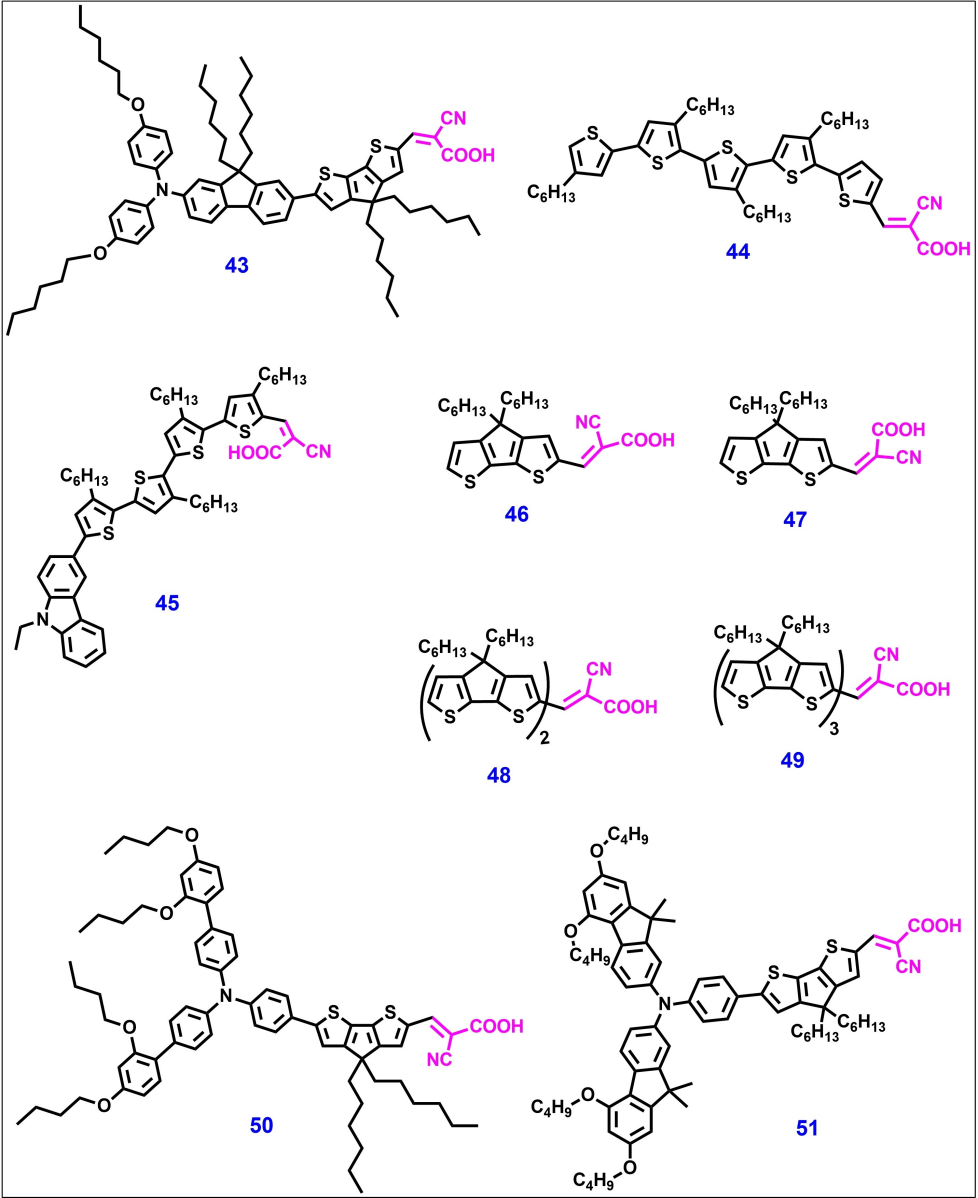
Chemical structures of metal‐free organic photosensitizers for ss‐DSSC applications.

Henry J. Snaith and co‐workers reported oligo(3‐hexylthiophene) (oligo‐3HT)‐based π–A (donor free) and D–π–A structured photosensitizers **44** and **45**, respectively, for ss‐DSSC applications and reported a PCE of 4.4 % (Figure 12). Both dyes have a similar molecular backbone structure, except for the *N*‐ethylcarbazole unit as a strong donor. Donor‐free dye **44** shows a better photovoltaic performance than dye **45** because of the higher steady‐state concentration of the oxidized sensitizer in the former, which improves the potential offset across the TiO_2_‐HTM heterojunction.^[143]^ Another series of “donor‐free” dyes **46**–**49** based on cyclopentadithiophene (CPDT) and functionalized with cyanoacrylic end groups was reported by Yue Hu et al. for both liquid‐state DSSCs and ss‐DSSCs.^[144]^ Among them, dyes **48** and **49** were shown to be effective photosensitizers for m‐TiO_2_ solar devices and delivered high PCE values with the liquid I^−^/I_3_^−^ (PCE of 6.7 %) electrolyte or [Co(bpy)_3_]^2+/3+^ (PCE of 7.3 %) electrolyte and spiro‐OMeTAD HTM (PCE of 3.9 %). In ss‐DSSCs, these dyes showed a remarkable J_SC_, broad absorption up to 900 nm, and a high molar absorption coefficient of 75000 M^−1^ cm^−1^.

For the first time in 2013, Licheng Sun and co‐workers reported a high PCE value using other alternative HTMs to spiro‐OMeTAD. As a result, an ss‐DSSC constructed with TPA‐based organic dye **50** and novel organic HTM afforded impressive PCEs of 5.8 % and 7.1 % under 1 and 0.46 sun, respectively. They demonstrated the possibility of developing low‐cost and high‐efficiency novel HTMs for ss‐DSSC applications based on organic dyes.^[145]^ Later, the same group reported a new organic D–π–A photosensitizer **51** and applied it to ss‐DSSCs using spiro‐OMeTAD as the HTM. They reported a high PCE of 6.1 % with a J_sc_ of 12.4 mA/cm^2^ under standard AM 1.5G illumination (100 mW/cm^2^).^[146]^ Compared to dye **50**, dye **51** displayed a broader and stronger absorption in the visible region and higher IPCE values. Theoretical studies of dyes **50** and **51** revealed that the latter has much lower re‐organization energy than that of the former and it may facilitate the hole‐hoping process among the multilayers of photosensitizers and between the photosensitizer and HTM, resulting into more efficient dye regeneration.

**Figure 13:**
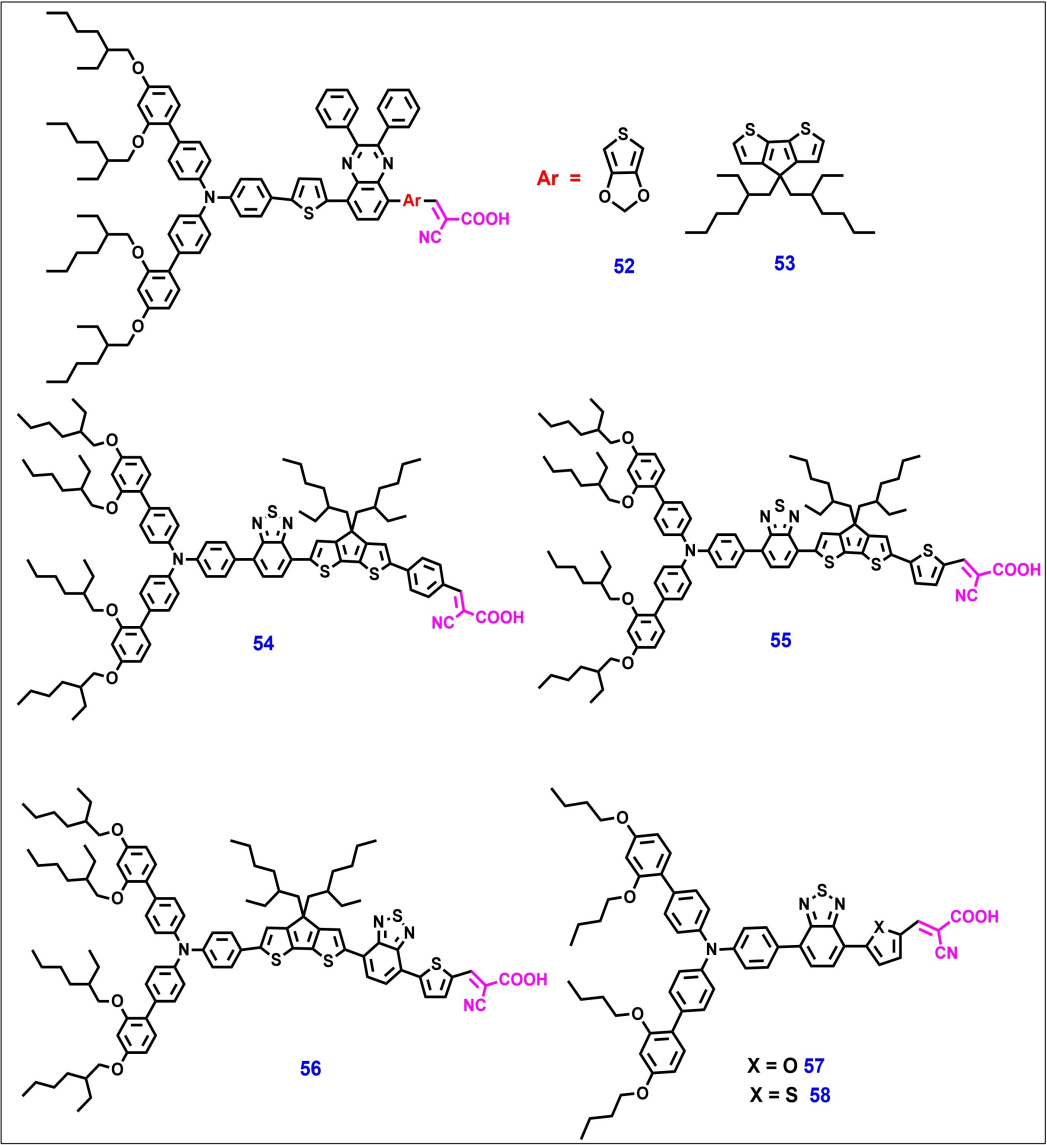
Chemical structures of metal‐free organic photosensitizers for ss‐DSSC applications.

Again, the same group designed and developed two novel quinoxaline‐based D–A‐π–A organic photosensitizers **52** and **53** (Figure 13) using 3,4‐ethylenedioxythiophene (EDOT) and CPDT as π‐spacer units, respectively. Both dyes have been studied for application in ss‐DSSCs with spiro‐OMeTAD as the HTM. A high PCE of 8.0 % was achieved for dye **56** under standard AM1.5 solar conditions. Under the same conditions, the state‐of‐the‐art organic D–π–A dye afforded a PCE of 7.3 %. Transient photovoltage decay measurements showed that, compared to ss‐DSSC devices based on dye **52**, those based on dye **53** afforded a longer electron lifetime and lower charge recombination losses. Regeneration of dye **53** by spiro‐OMeTAD can be more efficient than that of dye **52**; this has also been well explained by photo‐induced absorption (PIA) spectroscopy.^[147]^

In 2016, Michael Grätzel et al. reported a series of three novel D–A–π–A organic dyes **55**–**56** for application under ambient light and for building integrated ss‐DSSC applications. These dyes showed high molar extinction coefficient values of 5.65×10^4^, 6.66×10^4^, and 2.62×10^4^ and high absorption ranges of 552, 578, and 618 nm, respectively. The molecular engineering of these dyes was achieved by introducing a benzothiadiazole (BTZ) acceptor with a thiophene heterocycle unit, as well as by changing the position of BTZ on the conjugated backbone. Because of the high extinction coefficient value of dye **55**, it facilitated the formation of highly efficient thin‐film ss‐DSSCs with only a 1.3‐μm‐thick meso‐TiO_2_ layer and afforded a high photovoltaic performance of 7.51 %.^[148]^

In 2017, Lars Kloo et al. developed two novel organic dyes with D–A–π–A configuration (dye **57** and **58)**. An ss‐DSSC device constructed with these dyes afforded PCEs of 6.9 % and 5.2 %, respectively, with the spiro‐OMeTAD HTM.^[149]^ Compared to **59**, a well‐known dye,^[150]^ **57** and **58** afforded high‐performance ss‐DSSC devices owing to the presence of an extra triphenylamine moiety into the molecular back bone of the dyes. The photoinduced absorption spectroscopy (PIA) showed that these dyes exhibit efficient regeneration and high hole conductivity in the presence of the spiro‐OMeTAD HTM, indicating a pronounced charge transfer at the interfaces of the two systems.

**Figure 14:**
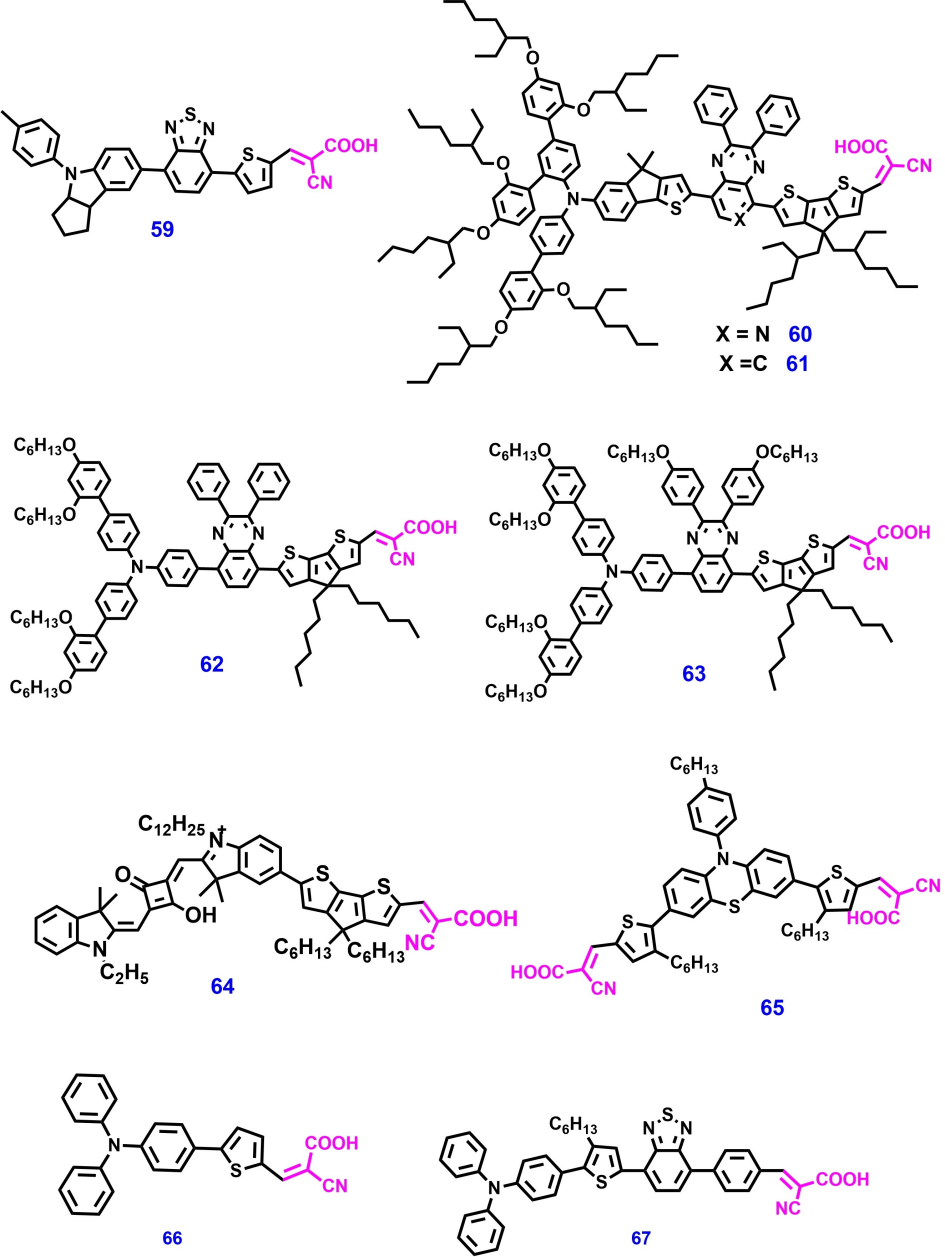
Chemical structures of metal‐free organic photosensitizers for ss‐DSSC applications.

In 2013, Z. Shen et al. reported two new metal‐free organic blue dyes **60** and **61** (Figure 14) for ss‐DSSCs. Their molecular structure consists of an indeno[1,2‐b]thiophene‐functionalized triphenylamine as the donor; 2,3‐diphenylpyrido[3,4‐b]pyrazine (PP) or 2,3‐diphenylquinoxaline (QT) as the auxiliary acceptor; and cyclopentadithiophene (CPDT as the π‐spacer unit. These novel dyes displayed very high molar extinction coefficients (2.7×10^4^ M^−1^ cm^−1^ for dye **60** and 6.3×10^4^ M^−1^ cm^−1^ for dye **61**) and broad absorption spectra in the visible region (555–700 nm). Hence, an ss‐DSSC device based on dye **60** as a photosensitizer and spiro‐OMeTAD as an HTM afforded very high PCEs of 7.81 % and 8.25 % under 1 sun and 0.5 sun light intensity, respectively. This is the first report to show that ss‐DSSCs with an organic blue dye have PCEs of >7.8 %.^[151]^

The main disadvantage of excitonic‐type solar cells is their very high voltage loss (V_loss_), which lowers the power conversion efficiency of solar cells. The molecular structure of photosensitizers and redox mediators can control the V_loss_ of solar cells via molecular engineering. On this basis, for the first time Michael Grätzel et al. reported metal‐free organic dyes **62** and **63** for ss‐DSSC applications and reported a high PCE of 11.7 %. The low V_loss_ of solar cells can be achieved by three important routes: (i) by minimizing the driving force of electron injection through appropriate molecular engineering of the dye, (ii) by decreasing dye regeneration overpotential by engineering redox mediators, and (iii) by avoiding interfacial electron recombination.^[152]^ Organic sensitizers with NIR absorption have gained much attention for ss‐DSSC applications, because of their ideal panchromatic light absorption (400–700 nm).

Among the several NIR‐absorption materials, squaraine‐based sensitizers are good candidates because of their characteristic strong absorption from the visible to the NIR region of the absorption spectrum. Amalie Dualeh et al. reported squaraine‐based dye **64** for ss‐DSSCs and reported a strong maximum absorption at 672 nm with a corresponding extinction coefficient of 250,000 M^−1^ cm^−1^.^[153]^ An ss‐DSSC device with spiro‐OMeTAD as the HTM and chenodeoxycholic acid (CDCA) as an additive displayed an outstanding PCE of 3.16 %. The main challenge with ss‐DSSC is to improve the PV performance in order to harvest maximum incident light energy. Co‐sensitization has been developed to improve the light harvesting efficiency and intramolecular energy transfer. However, this method decreases the dye loading due to limited surface area, which lowers PV performance.

Another alternative method is incorporation of thermally activated delayed fluorescence (TADF) material into ss‐DSSC device, which can improve the PCE by enhancing the amount of light absorption with increased dye loading. Seok et al. have studied the effect of the TADF material (4CzBN) as an energy donor and dye **65** as energy acceptor in fluorescence resonance energy transfer (FRET) based ss‐DSSC. The PV performances of ss‐DSSC fabricated by using dye **65** and different concentrations of TADF materials (0.0, 0.05, 0.10 and 0.15 wt%) were systematically analyzed. Among these FRET based ss‐DSSC device exhibited a remarkable PCE of 5.17 %, J_SC_ of 11.52 mA cm^−2^ at 0.10 wt%. This study suggested that the use of TADF materials as electron donor in FRET based ss‐DSSC is feasibly opening new insights into the development of highly efficient ss‐DSSC.^[154]^

Delices et al. developed a series of D–π–A structured triarylamine based dyes **66**, **67**, **77** and **78** for ss‐DSSC. Theoretical (DFT and TDFT) and experimental studies were performed to analyze the dyes molecular structure containing different D, π‐spacer, and A with optoelectronic characteristics including absorption, emission, HOMO and LUMO properties of dyes in organic and aqueous medium (water and acetonitrile). The HTM was deposited by in‐situ photoelectrochemical polymerization process in organic and aqueous medium. Among the four dyes, ss‐DSSC based on dye **67** exhibited a best PCE of 1.75 % high FF (0.57), V_oc_ (550 mV) and J_sc_ (5.6 mA/cm^2^).^[155]^

Incorporation of additional D or A units into the D–π–A structure, producing 2D–π–A or D–A’–π–A, respectively. These modifications lead to the broadening and shifting of dye absorption towards the NIR region. Robertson et al. developed oligo‐cyclopentadithiophene dyes **70**–**72** based on a combination of previously reported **68** and **69** dyes. The insertion of a thiophene unit between the cyclopenta[2,1‐b:3,4‐b]dithiophene (CPDT) and the cyanoacrylic acid acceptor group brings about significant changes in their optoelectronic properties. UV‐vis absorption studies revealed that these dyes exhibit a bathochromic shift together with a larger extinction coefficient value. DSSC device fabricated with dye **72** achieved a PCE of 5.88 % and 4.38 % with I^−^/I_3_^−^ and spiro‐OMeTAD electrolyte devices respectively.^[156]^

**Figure 15:**
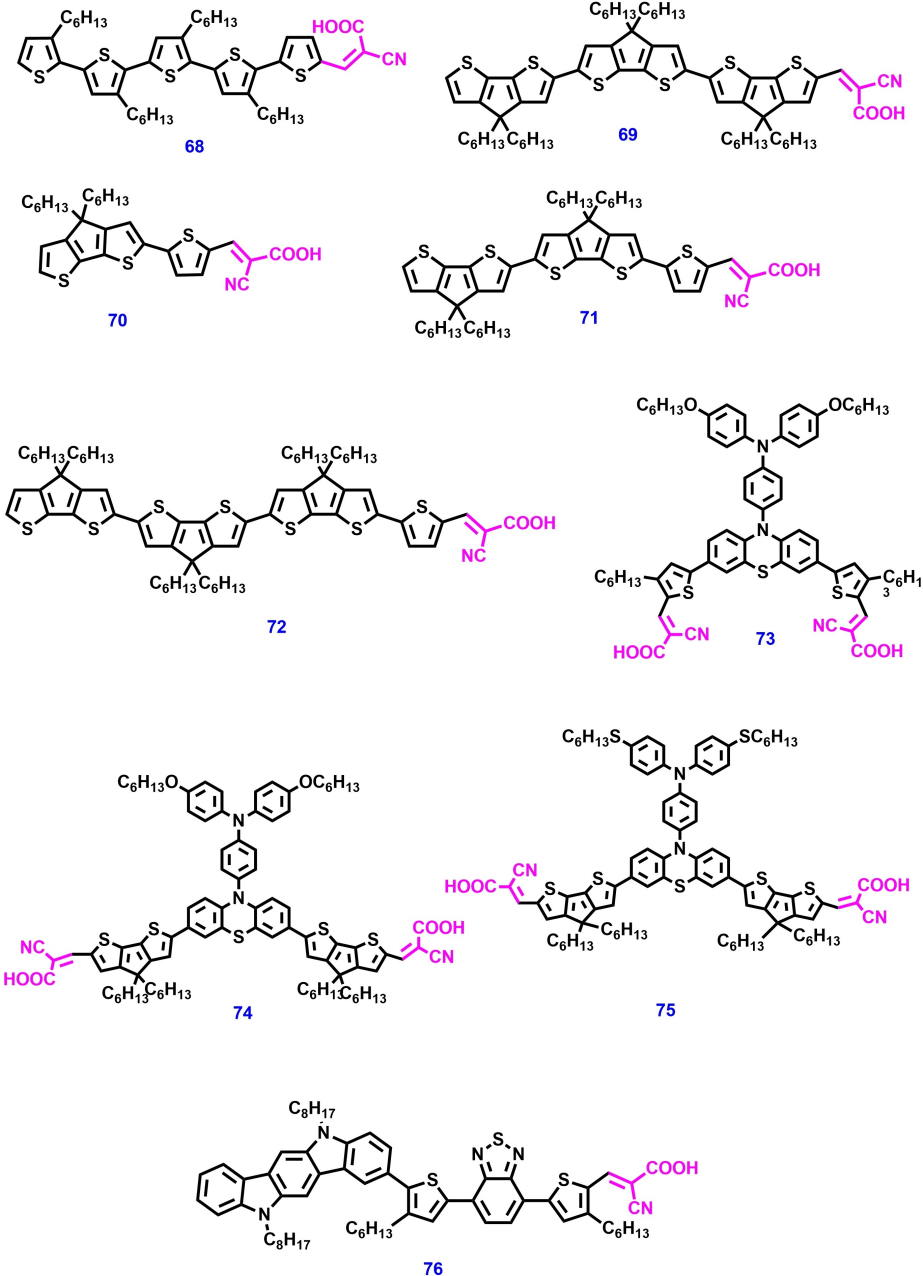
Chemical structures of metal‐free organic photosensitizers for ss‐DSSC applications.

In DSSC, double anchored dyes show numerous advantages including efficient dye packing and appropriate dye loading on a mesoporousTiO_2_ surface. Recently, Han et al. reported double anchored phenothiazine (PTZ) based dyes **73**–**75** with bulky *N*‐substituted triphenylamine units for DSSC. Among these dyes, the N‐substituent of 4‐(hexyloxy) phenyl substituted TPA (dye **73**) offered excellent steric matching to its double‐anchored PTZ dye. In addition, dye **73** facilitates the good and compact dye packing, as well as forming the integrated shelter layer on the TiO_2_ surface. As a result, DSSC with dye **73** exhibited a PCE of 10.47 % and 21.2 % under 1 sun and artificial light conditions respectively.^[157]^ The chemical structures of metal free organic dyes consisting of cyanoacrylic acid anchoring unit are shown in Figures 11 – 15 and their photovoltaic performance is tabulated in Table 2.

**Table 2:** Photovoltaic characterization of ss‐DSSC and DSSC devices with metal‐free organic dyes.

Dye	HTM	Additives	CE	J_SC_ (mA/cm^2^)	V_OC_ (V)	FF	PCE (%)	Ref
**34**	spiro‐OMeTAD	Li, tBP	Au	9.0	0.80	0.56	4.0	[137]
**35**	spiro‐OMeTAD	Li, tBP	Ag	7.2	0.86	0.73	4.5	[138]
**36**	spiro‐OMeTAD	Li, tBP	Ag	8.1	0.82	0.67	4.4	[138]
**37**	spiro‐OMeTAD	Li, tBP	Ag	9.7	0.88	0.71	6.1	[139]
**38**	spiro‐OMeTAD	Li, tBP, FK102	Ag	9.8	0.93	0.75	6.9	[140]
**39**	spiro‐OMeTAD	Li, tBP, FK102	Ag	11.6	0.80	0.61	5.6	[140]
**40**	spiro‐OMeTAD	Li, tBP, FK102	Ag	8.9	0.91	0.60	4.9	[140]
**38**	[Cu(tmby)_2_](TFSI)	Not reported	PEDOT	13.9	1.08	0.73	11.0	[141]
**41**	spiro‐OMeTAD	Li, tBP, FK209	Ag	8.5	0.81	0.71	4.9	[142]
**42**	spiro‐OMeTAD	Li, tBP, FK209	Ag	10.2	0.83	0.70	5.9	[142]
**43**	spiro‐OMeTAD	Li, tBP, FK209	Ag	9.7	0.87	0.75	6.3	[142]
**44**	spiro‐OMeTAD	Li, tBP	Ag	7.7	0.92	0.62	4.4	[143]
**45**	spiro‐OMeTAD	Li, tBP	Ag	6.9	0.70	0.58	2.8	[143]
**46**	spiro‐OMeTAD	Li, tBP, FK209	Au	6.5	0.85	0.67	4.0	[144]
**47**	spiro‐OMeTAD	Li, tBP, FK209	Au	7.8	0.72	0.59	3.5	[144]
**48**	spiro‐OMeTAD	Li, tBP, FK209	Au	10.9	0.73	0.47	3.9	[144]
**49**	spiro‐OMeTAD	Li, tBP	Ag	8.9	0.94	0.65	5.4	[145]
**50**	X3	Li, tBP	Ag	9.7	0.90	0.66	5.8	[145]
**51**	spiro‐OMeTAD	Li, tBP	Ag	12.4	0.78	0.63	6.1	[146]
**52**	spiro‐OMeTAD	Li, tBP	Ag	11.5	0.82	0.72	6.8	[147]
**53**	spiro‐OMeTAD	Li, tBP	Ag	12.3	0.87	0.75	8.0	[147]
**54**	spiro‐OMeTAD	Li, tBP, FK209	Au	5.31	0.91	0.71	6.98	[148]
**55**	spiro‐OMeTAD	Li, tBP, FK209	Au	11.0	0.90	0.76	7.5	[148]
**56**	spiro‐OMeTAD	Li, tBP, FK209	Au	5.67	0.77	0.65	5.8	[148]
**57**	spiro‐OMeTAD	LiTFSI	Ag	10.5	0.88	0.74	6.9	[149]
**58**	spiro‐OMeTAD	LiTFSI	Ag	9.6	0.87	0.62	5.2	[149
**59**	spiro‐OMeTAD	LiTFSI	Ag	7.0	0.67	0.49	2.3	[150]
**60**	spiro‐OMeTAD	Li, tBP, TeCA	Ag	9.1	0.80	0.65	4.7	[151]
**61**	spiro‐OMeTAD	Li, tBP, TeCA	Ag	12.9	0.83	0.73	7.8	[151]
**62**	[Cu(tmby)2]2+	Li, tBP	PEDOT	13.2	1.06	0.77	11.0	[152]
**63**	[Cu(tmby)_2_](TFSI)+	Li, tBP	PEDOT	13.8	1.07	0.79	11.7	[152]
**64**	spiro‐OMeTAD	Li, tBP, FK102	Ag	7.3	0.71	0.61	3.2	[153]
**65**	spiro‐OMeTAD	Li, tBP, FK102	Au	11.52	0.68	0.65	5.17	[154]
**66**	PEDOT	Li, tBP	Au	3.7	0.43	0.57	0.91	[155]
**67**	PEDOT	Li, tBP	Au	5.6	0.55	0.57	1.75	[155]
**68**	I^−^/I_3_^−^	Li, tBP	Pt	11.92	0.60	0.71	5.06	[156]
**69**	I^−^/I_3_^−^	Li, tBP	Pt	15.07	0.56	0.71	5.95	[156]
**70**	spiro‐OMeTAD	Li, tBP	Au	4.90	0.66	0.61	1.98	[156]
**71**	spiro‐OMeTAD	Li, tBP	Au	8.82	0.65	0.65	3.70	[156]
**72**	spiro‐OMeTAD	Li, tBP	Au	11.27	0.68	0.58	4.38	[156]
**73**	I^−^/I_3_^−^	Li, tBP	Pt	19.65	0.78	0.69	10.4	[157]
**74**	I^−^/I_3_^−^	Li, tBP	Pt	11.03	0.71	0.68	5.35	[157]
**75**	I^−^/I_3_^−^	Li, tBP	Pt	8.90	0.66	0.63	3.79	[157]
**76**	I^−^/I_3_^−^	Li, tBP	Pt	11.3	0.80	0.72	6.6	[159]
**77**	PEDOT	Li, tBP	Au	3.2	0.57	0.64	1.17	[155]
**78**	PEDOT	Li, tBP	Au	3.1	0.54	0.52	0.87	[155]
**79**	ZnO	–	Al	0.67	0.42	0.41	0.12	[158]
**80**	ZnO	–	Al	0.86	0.53	0.4	0.18	[158]
**81**	ZnO	–	Al	0.85	0.53	0.39	0.07	[158]
**82**	I^−^/I_3_^−^	Li, tBP	Pt	15.5	0.89	0.72	10.0	[159]
**83**	I^−^/I_3_^−^	Li, tBP	Pt	16.9	0.94	0.72	11.6	[159]
**84**	spiro‐OMeTAD	Sb, Li, tBP	Au	7.7	0.87	0.61	4.1	[160]
**85**	CuI	(C_2_H_5_)_3_HSCN	Au	14.1	0.55	0.54	4.2	[161]
**86**	spiro‐OMeTAD	Li	Ag	8.7	0.64	0.57	3.2	[162]

### 6.2 Organic dyes with carboxylic acid anchoring unit:

**Figure 16:**
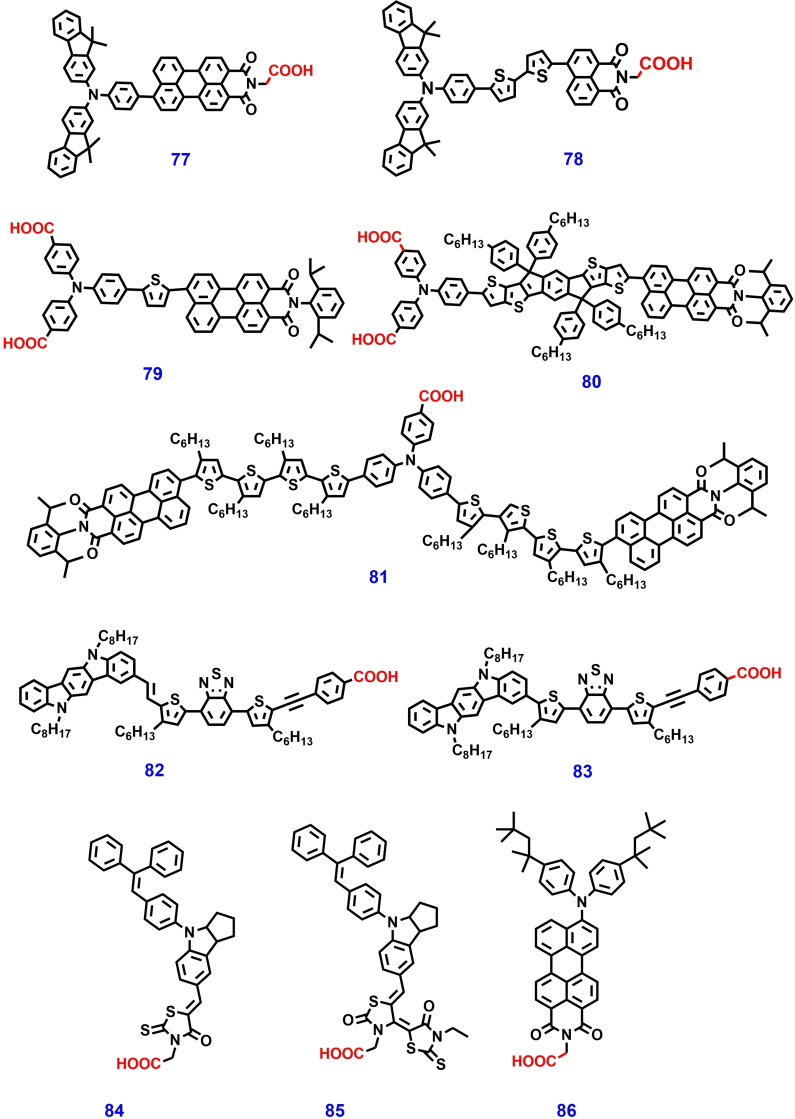
Molecular structure of organic photosensitizers consists of carboxylic acid anchoring for ss‐DSSC applications.

Xu et al. designed, and synthesized indacenodithieno[3,2‐b] thiophene (IDTT) based p‐type dyes **79**–**81** for p‐type ss‐DSSCs (Figure.16). These dyes consist of similar molecular units, including triphenyl amine that can act as an electron donor, perylene monoamide (PMI) acceptor, and carboxylic acid group that act as an anchoring site for metal oxide substrates. However, the linkers of the three dyes are different: dye **79** has a single thiophene unit, dye **81** consists of four alkylated thiophene units and dye **80** contains IDTT unit. IDTT is a ladder‐type, ‐conjugated unit with a rigid, coplanar structure, which has been widely used in variety of optoelectronic applications due its suitable optoelectronic properties. Dye **80** shows a maximum absorption at 554 nm and 550 nm in solution state and high extinction molar coefficient of 82100 L mol^−1^ cm^−1^ at 554 nm. As result, p‐ssDSSCs, constructed by using ZnO as ETM and dye **80** exhibited a higher PCE of 0.18 % than their counter part dye **73** (0.12 %) and dye **75** (0.07 %).^[158]^

Usually, the photosensitizers for DSSC consist of rigid and coplanar molecular units. To achieve high performance DSSCs, the molecular packing and orientation of the photosensitizer on m‐TiO_2_ surface plays an important role. Based on this, Zhang et al. designed three indolo[3,2‐b]carbazole‐based small molecular organic dyes **76**, **82** and **83**, which contains 5,11‐bis(2‐ethylhexyl)‐5,11‐dihydroindolo[3,2‐b]carbazole as an donor, 4,7‐bis(4‐hexylthiophen‐2‐yl)benzo[c] [1,2,5] thiadiazole as π‐spacer unit, 4‐ethynyl benzoic acid (EBA) and cyanoacrylic acid (CA) as acceptors. Finally, the D and A molecular units were linked by the Z‐type double bond and single bond. The thin film absorption of dye **83** on TiO_2_ film was redshifted as compared with those in solution state due to “side‐by‐side” accumulation J‐aggregation. However, the thin film absorption of dyes **82** and **76** were blue shifted leading to “head‐on‐head” accumulation H‐aggregates. Therefore, DSSC device based on dye **83** shows a significant PV performance of 11.6 % due to its dense packing and standing behavior on m‐TiO_2_ film.^[159]^

In 2005, for the first time, Lukas et al. reported metal‐free organic dye **84** for ss‐DSSC applications. The molecular engineering of dye **84** consists of using ethene‐1,1,2‐triyltribenzene as a strong donor, indoline unit as a π‐bridge/central unit, and rhodanine‐3‐acetic acid as an acceptor. This molecule has a high molar extinction coefficient of 55,800 M^−1^ cm^−1^ at 491 nm and shows broad absorption in the visible region. ss‐DSSC devices made using dye **84** as the photosensitizer and spiro‐OMeTAD as the HTM afford a PCE of >4 %. Therefore, indoline dye **84** is the first and produced alternative photosensitizer that can replace Ru‐based sensitizers for ssDSSCs.^[160]^ Another novel metal free organic dye **85** was reported by Akinori Konno et al. in 2007. The molecular structure of dye **85** was the same as that of dye **84**, except for the 3‐ethylrhodanine unit. This dye shows stronger absorption and a higher molar extinction coefficient value than those of well‐known Ru‐based dyes. A heterojunction ss‐DSSC device with an *n*‐TiO_2_/Dye **85**/p‐CuI configuration and copper iodide (CuI) act as an HTM afforded an efficiency of 4.2 %. Organic dyes strongly adsorb into TiO_2_ than metal‐based dyes and form non quenching aggregates, and hence, are more beneficial for ss‐DSSC applications.^[161]^

In 2009, Hagfelt et al. reported perylene‐based dye **86** for ss‐DSSC applications, which showed strong absorption from 400 to 700 nm.^[162]^ ss‐DSSCs with dye **86** and spiro‐OMeTAD as the HTM afforded a photocurrent of up to 9 mA cm^−2^ and a PCE of 3.2 %. Furthermore, this molecule was studied for application in conventional DSSCs using liquid I^−^/I_3_^−^ as the electrolyte and poor performance was noted. These studies show that liquid DSSC and ss‐DSSC devices have different injection and regeneration mechanisms. The incident photon‐to current conversion efficiency (IPCE) spectra of dye **86** in both liquid and solid electrolytes showed that the regeneration of the oxidized dye molecules by spiro‐OMeTAD is several orders of magnitude faster than that achieved in liquid iodide/tri‐iodide. In addition, the results of photoelectron spectroscopy and electrochemistry suggest that the sensitizer was able to efficiently inject into TiO_2_, when Li^+^ ions was present on the surface, while injection was very slow in the absence of Li^+^ ions or in the presence of solvent.^[163]^ The chemical structures of metal‐free organic dyes with carboxylic acid anchoring group are shown in Figure 16 and their photovoltaic performance is shown in Table 2.

## 7 Co‐Sensitization in ss‐DSSCs

Co‐sensitization has been identified as a new method to improve the efficiency of DSSCs.^[164]^ For high‐performance DSSCs, an ideal photosensitizer should have a high LUMO energy level for efficient electron injection into the semiconductor TiO_2_ and a sufficiently low HOMO energy level for effective regeneration of the oxidized dye molecules. However, the high‐efficiency DSSCs cannot be attained through sensitization of a single dye molecule alone, due to limited spectral absorption. Co‐sensitization, on the other hand, enables the use of multiple photosensitizers with appropriate energy levels, resulting in broader and stronger absorption and consequently, higher efficiency DSSCs.^[165]^ By combining two photosensitizers, one can efficiently mitigate the recombination issues, while other can extend the absorption spectrum beyond a certain limit.^[136]^ In recent years, through the co‐sensitization method, DSSCs have exhibited a very high PCE of >14 %.^[166]^ Therefore, the combination of molecular sensitizers is a crucial step towards fabricating highly efficient DSSCs.

**Figure 17:**
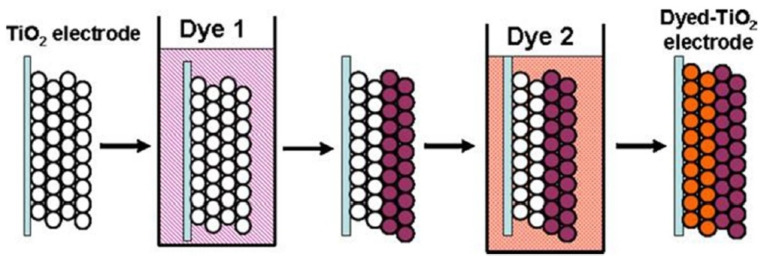
Schematic representation of the co‐sensitization in DSSC. Reproduced from Ref. [165] Copyright (2011), with permission from Elsevier.

Co‐sensitization is generally a two‐step process, where in the first step, proceeds sensitizing m‐TiO_2_ by the first dye is performed by using an appropriate solvent system within a fixed time to anchor the carboxylate tail on the TiO_2_ surface. This is followed by the sensitization of the second dye with an appropriate solvent system. Notably, this process is not supposed to affect the other dye in the co‐sensitized system. These deposition methods of dyes are called consecutive deposition, which are widely used in co‐sensitization. The schematic representation of co‐sensitization in DSSCs is shown in Figure 17.

**Figure 18:**
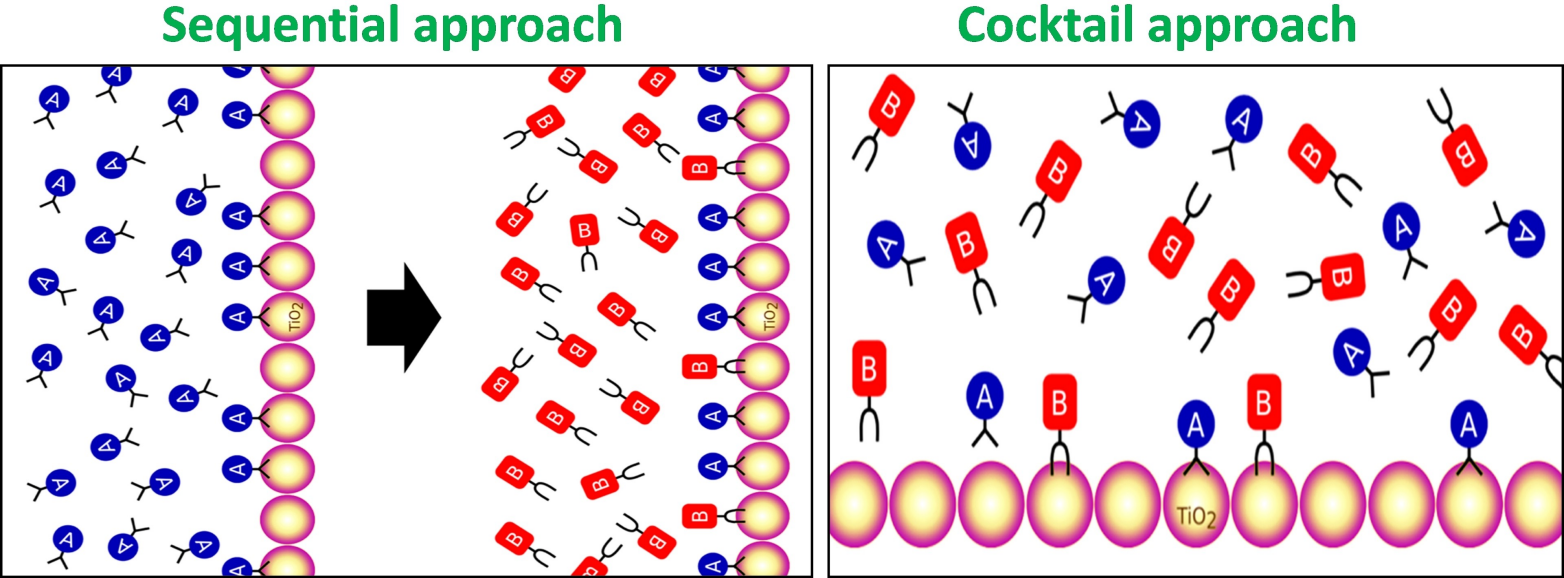
Sequential (left) and cocktail (right) co‐sensitization on TiO_2_ substrates. Reproduced from Ref. [167] Copyright (2019), with permission from American Chemical Society.

For co‐sensitization of dyes on the TiO_2_ semiconductor, two main avenues are usually employed: sequential and cocktail methods (Figure 18). The cocktail method is a one‐step co‐sensitization process that involves mixing different dyes in a single solvent, followed by dipping of TiO_2_ substrate into the resulting solution. The cocktail method supports the dye molecule in finding a comparatively unperturbed arrangement on the TiO_2_ surface. Although the cocktail method offers some advantages such as simple device fabrication, it generally gives less control on grouping chemically incompatible dyes, resulting in potential issues such as undesirable dye competition, unfavorable dye co‐sensitization ratios, and dye dissolution. In the sequential method, the semiconducting TiO_2_ surface is sensitized by one dye at a time. This method allows a greater level of control over dye heaping for every dye compared to that achieved with the cocktail method where multiple dyes are applied simultaneously.^[167]^ To achieve successful implementation of the co‐sensitization method, it is essential to carefully observe factors such as the sensitizing period of the dye, solvent selection, and appropriate deposition process. Enhancing absorption in ss‐DSSCs is critical for achieving high performance. While panchromatic sensitizers exhibit low absorption, making them nonideal for thin dye‐sensitized films, organic dyes tend to have narrow and stronger absorption bands. Co‐sensitization of complementary dyes has been shown to increase the photovoltaic response in liquid electrolyte cells, although it should be most useful for ss‐DSSCs in which the limited optical extinction is critical.

In 2011, Henry J. Snaith et al. reported the effect of surface energy of co‐sensitized molecules on the performance of ss‐DSSCs. They investigated the co‐sensitization of an organic sensitizer (dye **34)** showing visible absorption and that of a Zn‐phthalocyanine complex (dye **87**) exhibiting NIR absorbance and reported a considerably increased optical bandwidth in ss‐DSSCs with the spiro‐OMeTAD HTM. The co‐sensitized devices showed an enhanced PCE of 4.7 %, then the mono‐sensitized devices (PCE of 3.9 %). The spectroscopic experiments suggested that the resonant energy transfer from the visible to the NIR photosensitizer, leading to an overall enhancement in charge generation and performance of ss‐DSSCs.^[168]^ Surprisingly, the addition of an NIR sensitizer greatly increases the spectral behavior in the visible region, extending absorption to a broader range. This broad absorption enhances the photovoltaic performance of the existing visible sensitizers and potentially minimizes issues related to their aggregation, but more excitingly, it opens up new design criteria for a novel family of highly emissive, strongly absorbing visible sensitizers.

In 2012, Dualeh et al. reported the use of another NIR sensitizer in combination with squaraine‐based dye **67** and dye **38** respectively, this approach was found to broaden and intensify the light‐absorption spectra, resulting in improved performance of 4.4 %, surpassing that of devices based on single‐Dye‐sensitized.^[153]^ Furthermore, the electrochemical impedance spectroscopy (EIS) and transient photovoltage decay measurements showed that the charge transfer resistance for co‐sensitized devices increased compared to those containing only dye **67**. Series of ullazine‐based metal‐free organic dyes **88**–**91** were used for the first time in ss‐DSSC devices with the spiro‐OMeTAD HTM by Nazeeruddin *et al*. Among them dye **90** delivered the highest PCE of 4.95 %, which was further enhanced to 5.4 % through co‐sensitization with dye **38**.^[169]^ Lars Kloo et al. reported a novel D–π–A type blue dyes **92** and **93** by molecular engineering for efficient ss‐DSSCs *via* co‐sensitization method. These dyes were co‐sensitized with orange‐colored dye **38**, leading to an enhanced efficiency of 7.3 % and 7.5 % respectively.^[170]^ These results demonstrated that the photocurrent of ss‐DSSCs can be significantly improved by co‐sensitization, mainly owing to the wider light absorption range. In addition, the results from photo‐induced absorption (PIA) spectroscopy revealed that dye regeneration is more effective in co‐sensitized solar cells than mono‐sensitized DSSCs.

**Figure 19:**
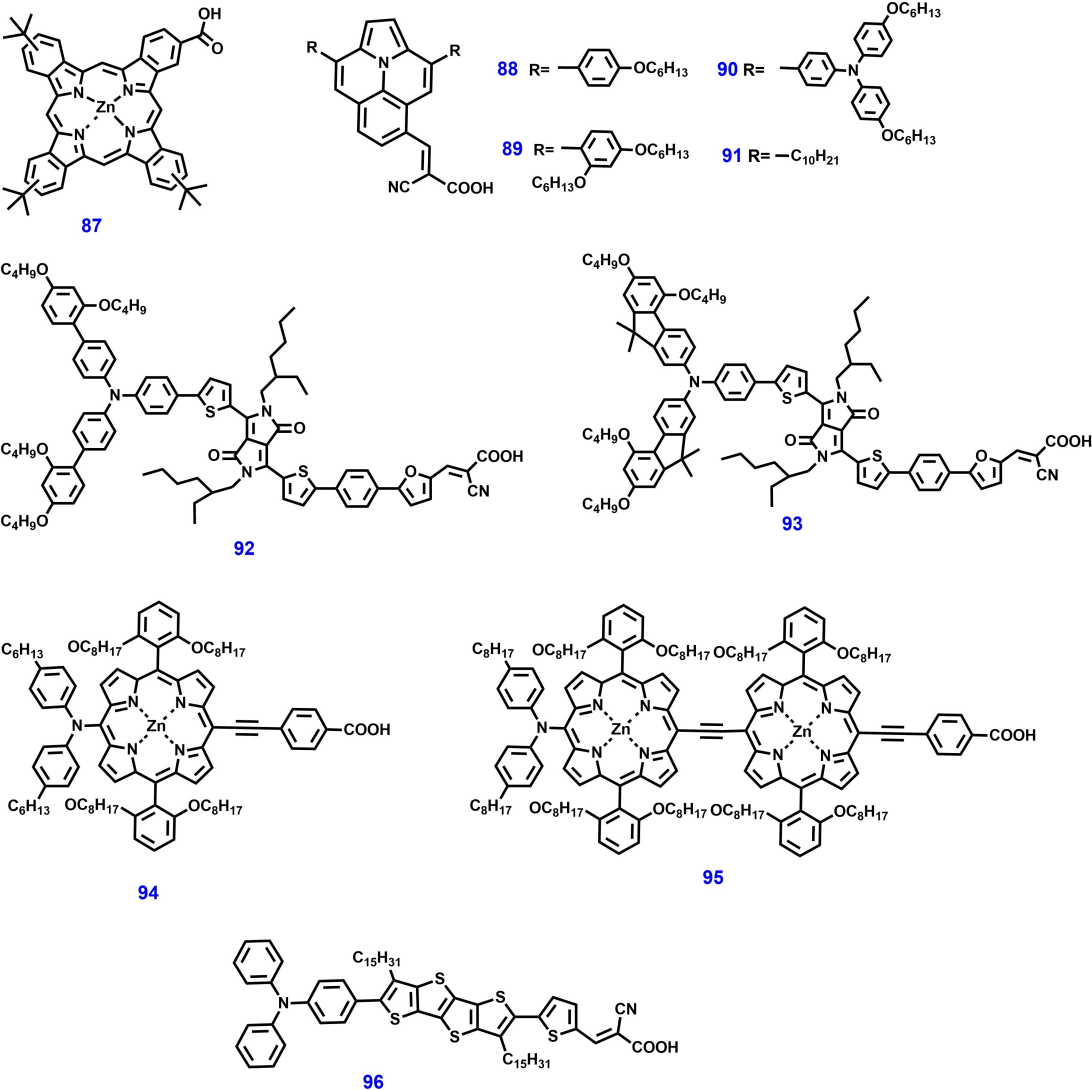
Chemical structures of dyes used in co‐sensitization method for ss‐DSSC applications.

A different co‐sensitization methodology using zinc porphyrin dye **12** and other suitable sensitizers such as zinc porphyrin dye **94** was reported by Yi et al.^[121]^ An ss‐DSSC device based on dye **12** and spiro‐OMeTAD as the HTM afforded a PCE of 4.8 % under 1 sun illuminations. Further, co‐sensitization with organic dye **41** resulted in an improved PCE of 6.4 %, which is more than 30 % higher to that achieved with mono‐sensitized devices. Robert P. H. Chang et al. later reported an ss‐DSSC device using an efficient mixture of dye **2** and two zinc porphyrin dyes **94** and **95**.^[171]^ A PCE of 7.8 % was successfully achieved with Cs_2_SnI_6_ as the HTM and a TiO_2_ nanostructured layer. Recently, there has been investigation into the possibility of using multiple dyes to extend the spectral range of solar spectrum. The findings indicated that the light harvesting efficiency across the solar spectrum is enhanced when porphyrin‐based dye **94** and organic dye **96** are used as complementary absorbers. This leads to an improved PCE of 8.53 % and long‐term stability when Cs_2_SnI_6_ was utilized as the HTM.^[172]^ The chemical structures of these dyes used for co‐sensitization of ss‐DSSCs are shown in Figure 19 and the corresponding photovoltaic parameters are shown in Table 3.

**Table 3:** Photovoltaic characterization of ss‐DSSC devices with metal free organic dye (Dye 1) and co‐sensitizer (Dye 2).

Dye1	Dye2	HTM	Additives	CE	J_SC_ (mA/cm^2^)	V_OC_ (V)	FF	PCE (%)	Ref.
**84**	**87**	spiro‐OMeTAD	Li, tBP	Ag	9.4	0.80	0.60	4.7	[168]
**35**	**88**	spiro‐OMeTAD	Li, tBP, FK102	Ag	8.5	0.74	0.66	4.19	[169]
**35**	**89**	spiro‐OMeTAD	Li, tBP, FK102	Ag	9.7	0.75	0.66	4.88	[169]
**35**	**90**	spiro‐OMeTAD	Li, tBP, FK102	Ag	10.6	0.80	0.64	5.40	[169]
**35**	**91**	spiro‐OMeTAD	Li, tBP, FK102	Ag	7.5	0.73	0.68	3.98	[169]
**50**	**92**	spiro‐OMeTAD	Li, tBP, FK102	Ag	12.41	0.88	0.67	7.3	[170]
**35**	**93**	spiro‐OMeTAD	Li, tBP, FK102	Ag	12.85	0.85	0.69	7.5	[170]
**96**	**94**	Cs_2_SnI_6_	Li, tBP	Pt	17.79	0.67	0.69	8.53	[121]
**2**	**95**	Cs_2_SnI_6_	Li, tBP	Pt	18.6	0.62	0.68	7.8	[171]
**2**	**96**	Cs_2_SnI_6_	Li, tBP	Pt	10.36	0.71	0.72	5.39	[172]
**35**	**92**	spiro‐OMeTAD	–	Ag	12.85	0.85	0.69	7.5	[173]

## 8 ss‐DSPV under Indoor Light Conditions

**Figure 20:**
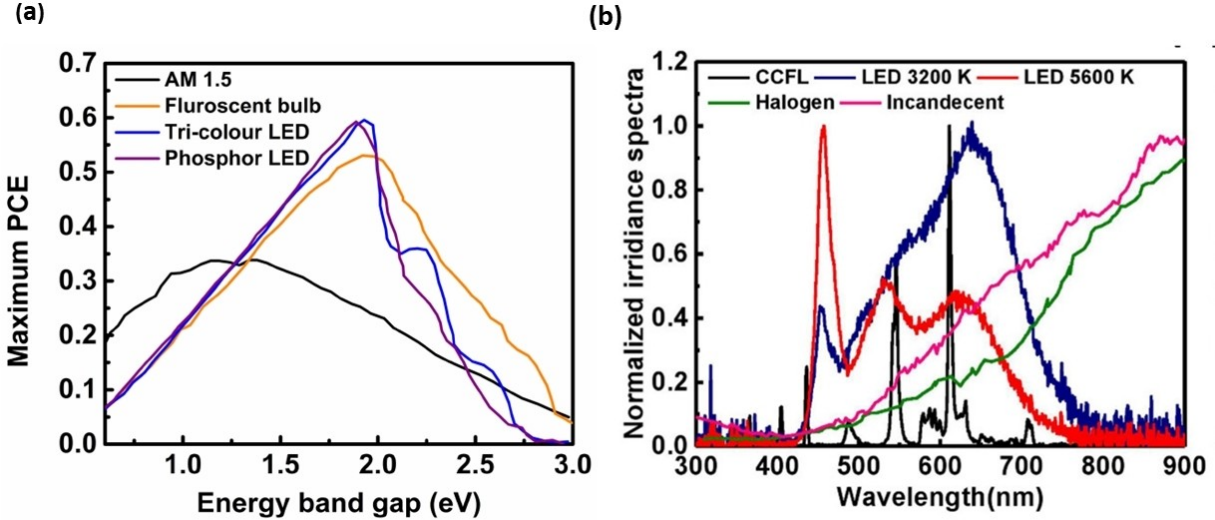
(a) Comparison spectra between the maximum PCE and band gap values of multiple sensitizers under different illuminating agents. (b) Irradiance spectra of different artificial/indoor light sources.^[183]^ Reproduced from Ref. [183] Copyright (2015), with permission from MDPI journal.

A photosensitizer used for normal solar cells should have broad and extensive absorption spectra because the irradiance spectrum of sunlight has cover from high‐energy ultraviolet (300 nm) to the lower‐energy near infrared (NIR) (1800 nm) region.[^21^, ^174^, ^175^, ^176^, ^177^] Therefore, to minimize any unpredicted energy loss in indoor solar cells owing to thermalization and non‐absorption of light, the photosensitizers used for indoor solar cell applications should have narrow absorption bands.^[178]^ Theoretical calculations have shown that the optimal band gap of sensitizers for indoor and outdoor solar cell applications is approximately 1.9 and 1.35 eV, respectively (see Figure 20a).^[179]^ In the last decade, various types of photosensitizers have been widely studied under indoor‐light illuminations to develop highly efficient, flexible, durable, and most stable indoor solar cell devices.[^36^, ^180^, ^181^, ^182^]

Usually, different light sources such as cold cathode fluorescent lamps (CCFL), light emitting diodes (LEDs) (LED 5600 K and LED 3200 K), halogen, and incandescent lamps are used for measuring the indoor performance of ss‐DSPVs. As per Figure 20b, the irradiance spectra of LED 3000 K, LED 5600 K, and CCFL based lights are mainly covering the visible region, which provides a great opportunity of developing indoor photovoltaics employing wide band gap photoactive materials. In contrast, the irradiance spectra of incandescent and halogen lamps cover a wide range of visible wavelengths and extends to the near infrared (NIR) ranges (300–900 nm). In addition, the material of indoor photovoltaics designed for harvesting the light energy of halogen lamp and incandescent lamps needs to consider the spectra response up to NIR range. Therefore, currently LEDs and fluorescent lamps have attracted significant interests and dominate over incandescent and halogen lamps in the market.^[183]^

For the first time, Grätzel et al. reported a dye‐sensitized photovoltaic (DSPV) device based on three‐layered structures comprising nanosized TiO_2_, zirconium dioxide, and carbon for indoor applications. This three‐layered device consisting of N719 (dye **2**) Ru‐based dye as a photosensitizer was used on nanocrystalline solar cells under the Philips TLD 840 fluorescent lamp (low light levels (<300 lux)) and resulted in an equal or higher photovoltaic performance than that of the commercially available amorphous silicon module.^[184]^ Later, several research groups across the globe reported a liquid electrolyte‐based DSPV for indoor applications by using different types of sensitizers such as metal complexes, porphyrin, and metal‐free organic small molecules for indoor applications.^[182]^

Because of the increasing demands for portable electronic devices for wireless sensor networks (WSNs) and IoT applications, ss‐DSPVs are gaining attention for their efficient working under indoor (ambient) low‐light conditions. Although the highest PCE reported with conventional solar photovoltaics was 36.2 % under low‐light conditions, the stability issue remains a major concern in indoor photovoltaics.^[185]^ Sublimation of iodine ions and leakage and evaporation of organic solvents are two major limitations in conventional iodine‐based DSPVs. Therefore, a better alternative is needed for successful implementation of highly stable devices. With this aim, researchers have been focusing on the design and synthesis of solid‐state redox couples that can operate under significantly low‐intensity light conditions with long‐term stability.

**Figure 21:**
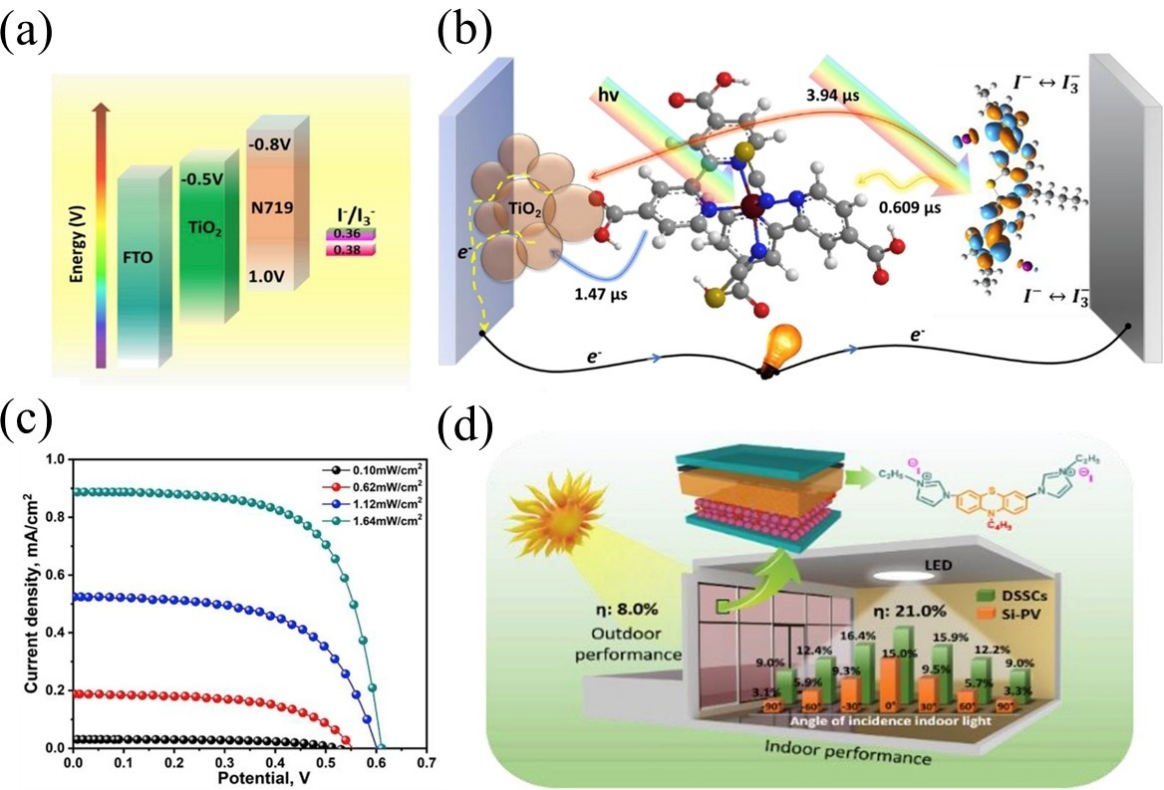
(a) Energy level diagram of N719 dye and ETRC electrolyte based ss‐DSSC. (b) Schematic representation of the energy transfer possibilities between ETRC (donor) and N719 dye (acceptor) molecules, (c) J–V characteristics of ETRC electrolyte based ss‐DSSC under various light intensities, and (d) Overall device performance of ss‐DSSC under various angle of indoor light illumination conditions.^[187]^ Reproduced from Ref. [187] Copyright (2015), with permission from Elsevier.

Efficiency of over 10 % was realized using the PAN/EC/PC:Pr_4_NI (polyacrylonitrile/ethylene carbonate/propylene carbonate:tetra propyl ammonium iodide) polymer gel electrolyte under low‐light condition (3 mW/cm^2^), which is 236 % higher than that achieved with the device PCE under 1 sun condition.^[186]^ High ionic conductivities of the polymer gel electrolyte (2.6 mS/cm at 25 °C and 4.8 mS/cm at 60 °C) were responsible for increment in the photocurrent, which greatly depends on the diffusion limitation of redox mediators. Jayraj et al. reported the synthesis of 3‐ethyl imidazolium iodide as an energy‐transfer redox couple (ETRC) to harvest the low light intensity for ss‐DSPVs and obtained a high PCE of 21.08 %, which is 28 % higher than that of the commercial Si solar cells under 1.64 mW/cm^2^ intensity.^[187]^ The dual functionality of this electrolyte advancing both energy transfer mechanism and act as redox couple (Figure 21 a,b) which offers the prospect of increasing photocurrent and efficient dye regeneration. Figure 21c represents the J–V curve of ss‐DSSC under various light intensities measured from 0.1 to 1.64 mW/cm^2^. The high device performance was realized for the device placed at 0‐degree angle of incidence than other angles (Figure 21d).

**Figure 22:**
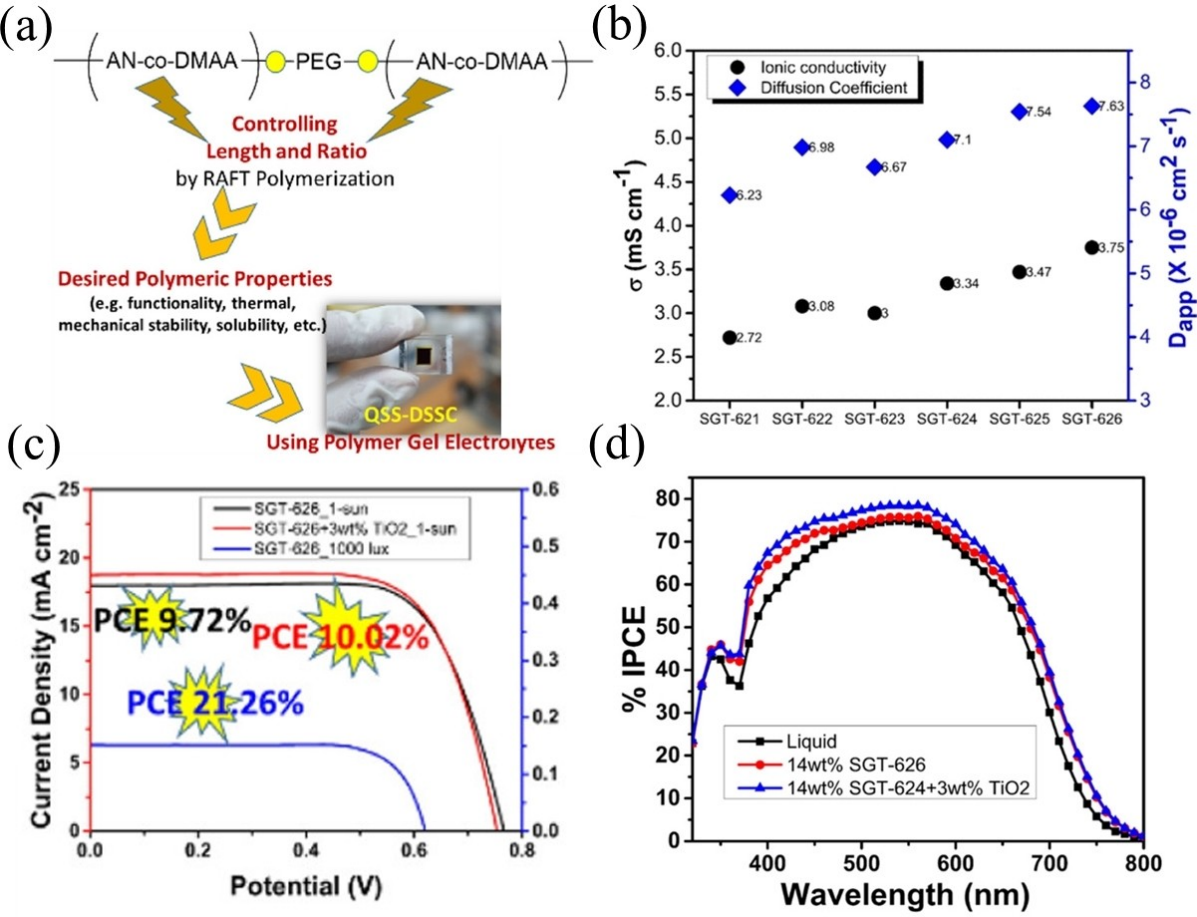
(a) Schematic representation of preparation of PEG‐Functionalized ABA Triblock Copolymers for Quasi‐Solid‐State Dye‐Sensitized Solar Cells. (b) Comparison of ionic conductivities and diffusion coefficients of SGT‐621, SGT‐622, SGT‐623, SGT‐624, SGT‐625, and SGT‐626. In all six triblock polymers the ionic conductivities and diffusion coefficients increase, while increasing molecular weight. (c) J–V characterization of DSSC device fabricated with SGT‐626 triblock copolymer measured under 100 mW cm^−2^ (AM 1.5G) illumination and artificial light (1000 lux) conditions. (d) IPCE spectra of liquid DSSC, QSS‐DSSC with 14 wt% SGT‐626 and QSS‐DSSC with 14 wt% SGT‐626+3 wt% TiO_2_. Reproduced from Ref. [188] Copyright (2015), with permission from American Chemical Society.

Masud et al. prepared 14 wt% SGT‐626 polymer gel electrolyte through reversible addition‐fragmentation chain‐transfer (RAFT) polymerization (Figure 22a) and utilized it as a solid‐state polymer electrolyte in DSPVs. It exhibited a high PCE of 21.26 % under white‐LED conditions (1000 lux), which was higher than the device efficiency of 19.94 % obtained for liquid‐state DSPVs.^[188]^ The ionic conductivity and the diffusion co‐efficient of polymers increases by increasing the molecular weight and decreasing the acrylonitril/N,N‐dimethylacrylamid ratio of the triblock copolymer (Figure 22b). It may be attributed to the lower‐end group which affects the larger polymeric chains and enhanced the dissociation of LiI salt facilitated by high DMAA content in the triblock copolymer. Figure 22 c–d represents the J–V and IPCE characteristics of ss‐DSSC made with SGT 626 and with the TiO_2_ fillers. It was noted that the solid‐state device with TiO_2_ filler exhibits a high current density in J–V towards enhancing the device PCE and it was further supported with the quantum efficiency enhancement in the IPCE spectrum.

Shanmuganathan Venkatesan et al. reported the effects of a metal nanofiller zinc oxide (ZnO) in cobalt‐based polymer gel electrolyte (PGE) prepared using poly(vinylidene fluoride‐co‐hexafluoropropylene) (PVDF‐HFP) for application in ss‐DSPVs.^[176]^ Approximately 4 wt% ZnO was used as the optimized concentration of nanofillers in quasi solid‐state DSPVs and realized a device efficiency of ~20.11 % under 200 lux of T5 fluorescent light intensity. Though the presence of ZnO nanofillers reduced the ionic diffusivity and conductivity of polymer gel electrolytes, they increase the efficiency of the electrolytes because of high charge transport resistance (R_ct_) and reduced chemical capacitance (C_μ_). The stability test of ss‐DSPVs without nanofillers showed that approximately 98 % of its initial device efficiency was retained even after 1044 h under room‐light illumination (200 lux at 35 °C). In devices with ZnO nanofillers, the PCE increases with aging; even after 1044 h of illumination, the PCE remained higher than its initial value (with a stability of more than 100 %).

**Figure 23:**
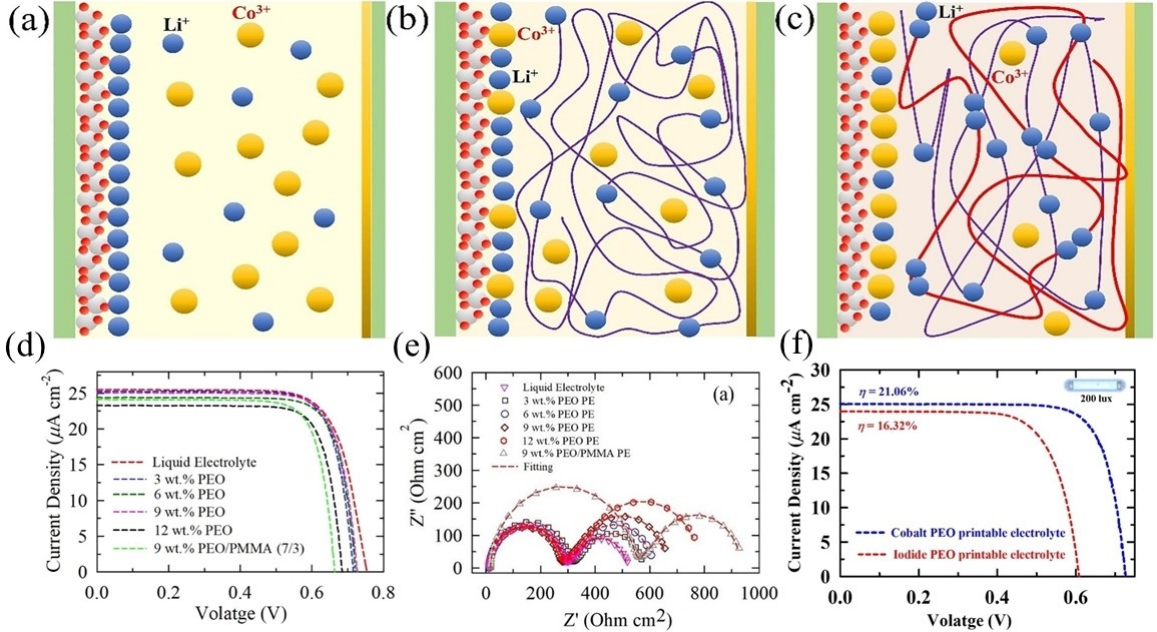
(a) Schematic model of Li^+^ and Co^3+^ distribution for DSSCs using (a)LE, (b) PEO‐PE and (c) PEO/PMMA PE, (d) J–V curve and (e) EIS‐Nyquist plot of DSSCs with cobalt LE and PEs with various concentrations of PEO and 9 wt% PEO/PMMA, and (f) High performance cobalt polyethylene oxide (PEO) printable electrolyte for DSSCs operating under 200 lux T5 light illumination. Reproduced from Ref. [190] Copyright (2015), with permission from Elsevier.

Moreover, polyethylene oxide (PEO)/polyvinylidene fluoride (PVDF) with TiO_2_ as a nanofiller can be used as a polymer electrolyte (PE) in ss‐DSPVs and a PCE of up to 20.63 % can be achieved under 600‐lux illumination.^[189]^ While preparing the module, a four‐strip module cell with the N719 dye showed 11.38 % of device efficiency and a rectangular module cell using the Z907 dye exhibited a device efficiency of 12.23 % under 200 lux. After the aging process, ss‐DSSCs based on N719 and Z907 dyes retained ~95 % and 97 % of the initial device PCE after 1000 h of illumination (200 lux; 35 °C). Usage of cobalt polyethylene oxide‐based printable electrolytes (Co‐PEO PE) resulted in increased PCE of 21.06 % with the Y123 dye under 200‐lux illumination (67.56 μW/cm^2^).^[190]^ In this study, the mechanism proposed on the distribution of Li^+^ and Co^3+^ ions in liquid and polymer electrolytes are shown in Figure 23a–c. In the electrolyte containing cobalt redox couple, the key cations are Li^+^ and Co^3+^ redox couple, and the competing adsorption of those cation ions will determine the electrical double layer and photoelectrodes. Since Li^+^ is smaller, it can easily penetrate into TiO_2_ films than cobalt ions. The high concentration of the Li^+^ ions on the photoelectrode leads to a more positive electric double layer which results in a higher repulsion force compared to the cobalt ion (Co^+3^) and thereby reduces the recombination kinetics. On the other hand, the PEO contains ether group (−C−O−C), and both ether and carbonyl (C=O) groups are involved in PMMA which contain a lone pair of electrons that have a strong tendency to coordinate with alkali metal ions such as Li^+^ (Figure 23b–c) Hence, the concentration of Li^+^ ions will decrease at the presence of the polymers and the Co^+3^ ions have a higher prospect to contact with the photoelectrode which resulting in a lower device performance. Figure 23d–e represents the J–V curve and Nyquist plot of ss‐DSSCs made with various cobalt based liquid and polymer electrolytes. It reveals the better performance for the device with 9 wt% PEO which is nearly equal to the conventional LE based DSSC. The device performance varied from 16.32 % (for iodide PEO electrolyte) to 21.06 % (Figure 23f) with cobalt PEO based printable electrolyte under low‐intensity light conditions (200 lux T5) along with the sub‐module efficiency of 12.58 %.

**Figure 24:**
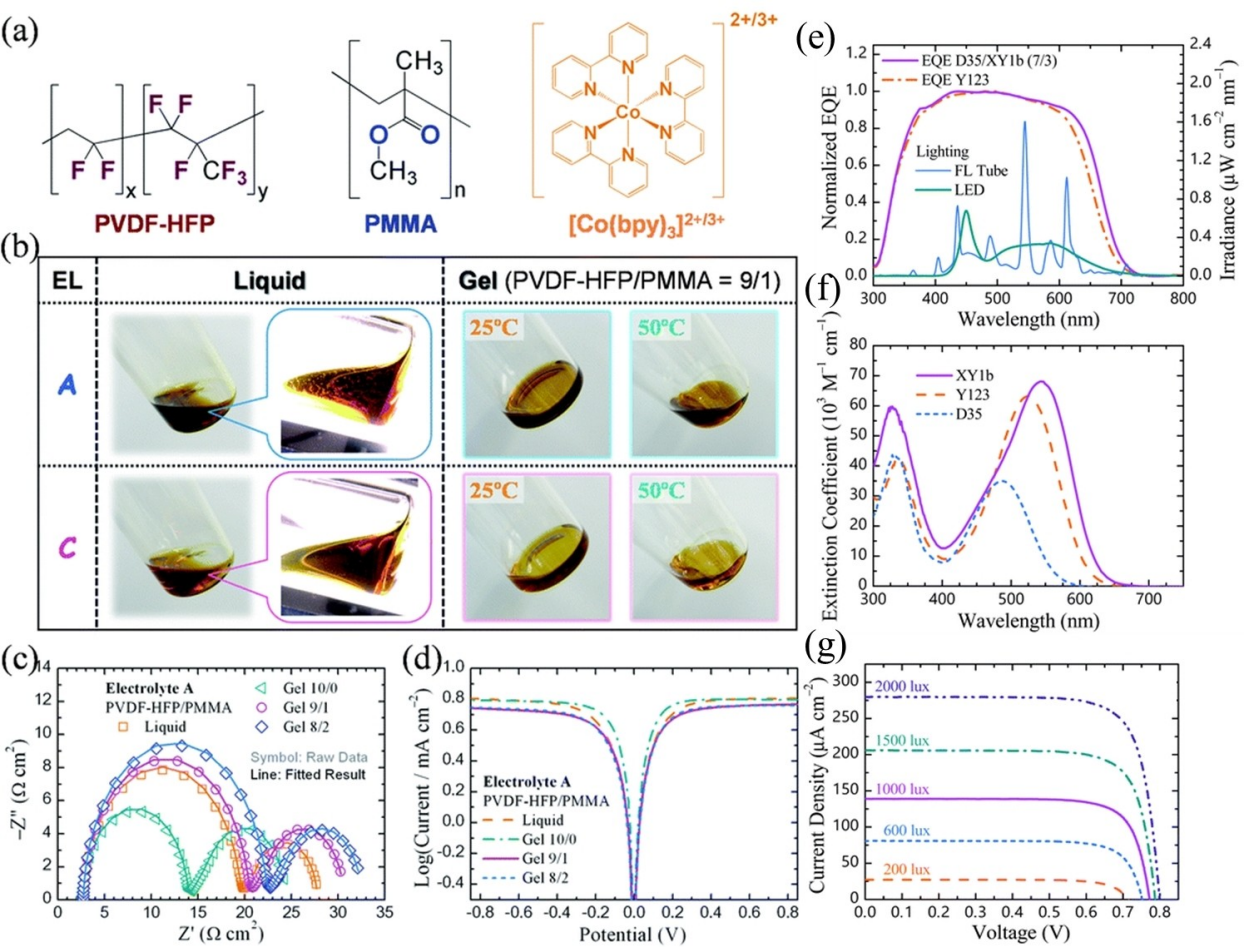
(a) Chemical structures of PVDF‐HFP, PMMA, and [Co(bpy)_3_]^2+/3+^ redox couples. (b) Photographs of the liquid and quasi‐solid‐state electrolytes: electrolyte A (0.22 M Co(II), 0.05 M Co(III), 0.1 M LiClO_4_, 0.2 M tBP) is same to the initial liquid electrolyte, while electrolyte C consists of 0.11 M Co (II), 0.025 M Co (III), 0.1 M LiClO_4_, and 1.2 M tBP. (c) Nyquist analysis (at 0 V) and (d) Tafel polarization curves of the studied electrolytes prepared using the starting composition. (e) Irradiance spectra of the fluorescent (FL) and LED lamp at 200 lux, and normalized EQE spectra of the ss‐DSPV cells using Y123 and D35/XY1b sensitization conditions. (f) UV‐vis absorption spectra of XY1b, Y123 and D35 in dichloromethane solution. (g) J–V curves of ss‐DSPV cells with co‐sensitization (D35/XY1b=7/3) under different fluorescent lighting Reproduced from Ref. [191] Copyright (2015), with permission from Royal Society of Chemistry.

Lee et al. reported a PCE of approximately 25.3 %, the highest value to date, for ss‐DSPVs under low‐light conditions (1000 lux; P_in_=329 μW/cm^2^) using Co complex‐PVDF‐HFP/PMMA gel electrolyte and dye **35**/XY1b co‐sensitized device structure by maintaining 96 % of the initial device efficiency even after 2000 h. The chemical structures of electrolyte materials used in this study are shown in Figure 24a. The quasi‐solid‐state gel‐type electrolytes were prepared with PVDF‐HFP and PMMA polymer blends in blend ratio of 10/0, 9/1, and 8/2 wt% respectively. Figure 24b exhibited photographs of liquid and gel type electrolytes based on the 9/1 blend ratio. The gel type electrolytes show several fluid characteristics at 50 °C but it shows their gel type behavior without apparent fluidity at 25 °C. Therefore, the gel‐type electrolytes were introduced into the DSSC device after preheated and appropriate characterization were performed after they cooled down. The charge transfer resistance (R_ct_) at the Pt‐electrode/electrolyte interface were analyzed by the EIS measurement (Figure 24c). The R_ct_ value of the liquid electrolyte A is 8.32 Ω cm^2^, and after the gelation only by the PVDF‐HFP with blend ratio of 10/0, the smaller R_ct_ of 5.58 Ω cm^2^ is obtained. However, the gel electrolyte prepared with 9/1 and 8/2 blend ratios of PVDF‐HFP shows higher R_ct_ value of 8.78 and 9.72 Ω cm^2,^suggesting that a higher content of PMMA causes a larger R_ct_. Similarly, Tafel polarization curves (Figure 24d) of gel electrolyte with a blend ratio of 10/0 reaches plateaus at lower potentials compared to other gel electrolyte systems, indicates a smoother redox reaction at the electrode/electrolyte interface and is consistent with the corresponding smallest R_ct_ value. The ss‐DSPV cell (dye **39**) shows a broad EQE spectrum, which utilizes light beyond 600 nm is incompletely, especially the second strongest emission peak at 612 nm of FL spectrum. Therefore, the co‐sensitization (dye **35**/XY1b) is implemented to increase the light absorption property (Figure 24e). The dyes XY1b and dye **35** show maximum absorption at a longer wavelength 547 nm and 484 nm respectively. The co‐sensitization of XY1b and D35 dyes can harvest photons in the short‐ and long wavelength regions, results in a broader EQE spectrum (Figure 24f). J–V characteristics of ss‐DSSC under various FL and LED‐light illuminations are illustrated in Figure 24g.^[191]^

**Figure 25:**
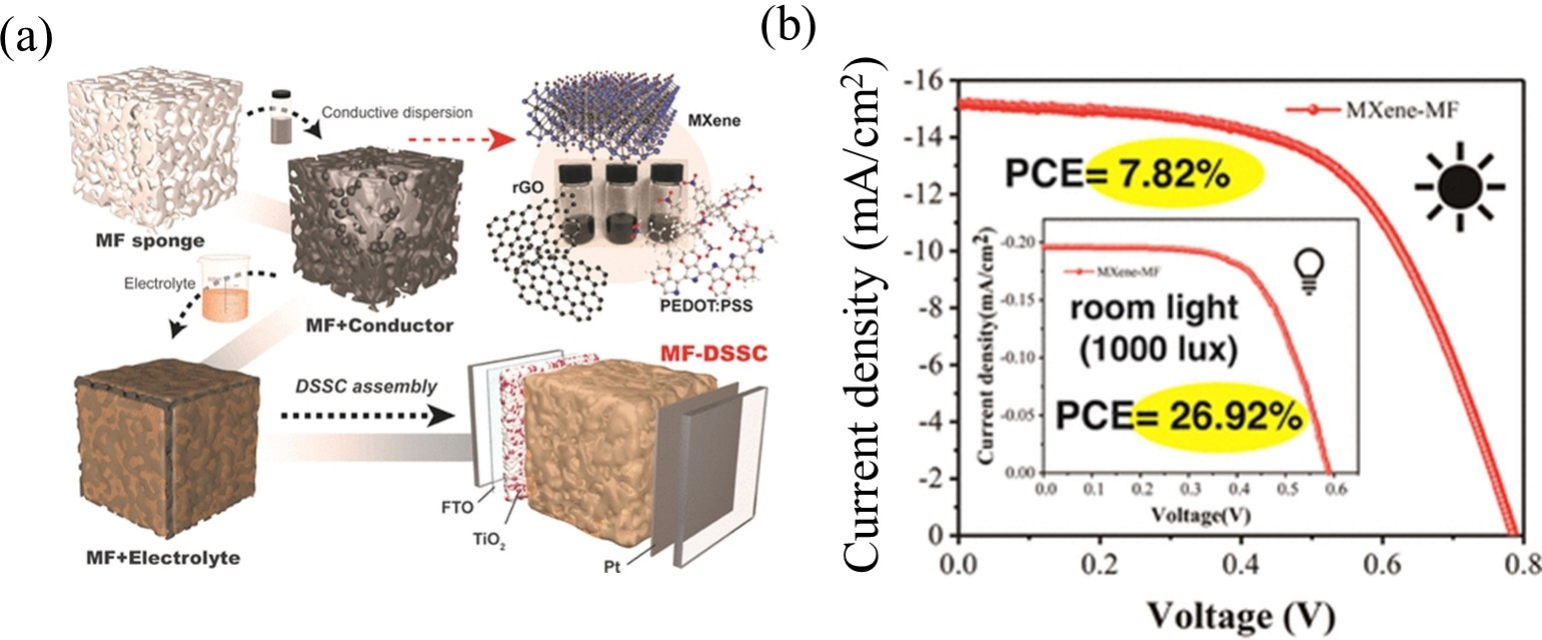
(a) Schematic representation of the modified Melamine Formaldehyde (MF)−DSSC with various conductive fillers, and (b) J–V curves of MF sponge quasi‐solid electrolyte DSSCs modified with various conductive fillers under 1‐sun and indoor light conditions (1000 lux). Reproduced from Ref. [194] Copyright (2023), with permission from American Chemical Society.

In 2013, Byrne et al. reported an ss‐DSPV device for indoor applications using a succinonitrile‐based material as a solid‐state electrolyte and N719 (dye **2**) as a photosensitizer and achieved energy conversion efficiencies of 6.3 % and 5.6 %, respectively. This ss‐DSSC device was tested under the illumination of a fluorescent bulb (200 lx) having the 1.63 μW power‐harvesting capability under ambient light.^[192]^ Nishide et al. developed an ss‐DSPV device by using organic indoline‐based dyes as a photosensitizer and highly reactive nitroxide radical materials as the electrolyte. Their device delivered a power‐conversion efficiency of 10.1 % under 1 sun illumination and also retained the same output performance even under indoor‐lighting conditions.^[193]^ At present, Zhicheng et al. reported a high PCE of 26.92 % under indoor light condition (1000 lux) using a melamine formaldehyde sponge electrolyte in ss‐DSSC and realized the stability of c.a. 90 % after 1000 h.^[194]^ In this work, the mighty sponge was soaked in three type of various conducting fillers by immersing in electrolyte and prepared the quasi‐solid electrolyte such as MXene‐MF, rGO‐MF, and PEDOT:PSS‐MF (shown in Figure 25a). The obtained composite sponge electrolyte layer was employed between the photoanode and the counter electrode to assemble three quasi‐solid electrolyte DSSCs. The J–V curve of *qs*‐DSSC assembled with MXene‐MF electrolyte system was shown in Figure 25b which reveals the higher device performance under indoor/1‐sun conditions due to the existence of conducting fillers in electrolyte system. The enhancement in J_sc_ and FF under indoor light condition, is possibly due to MF fillers.

**Figure 26:**
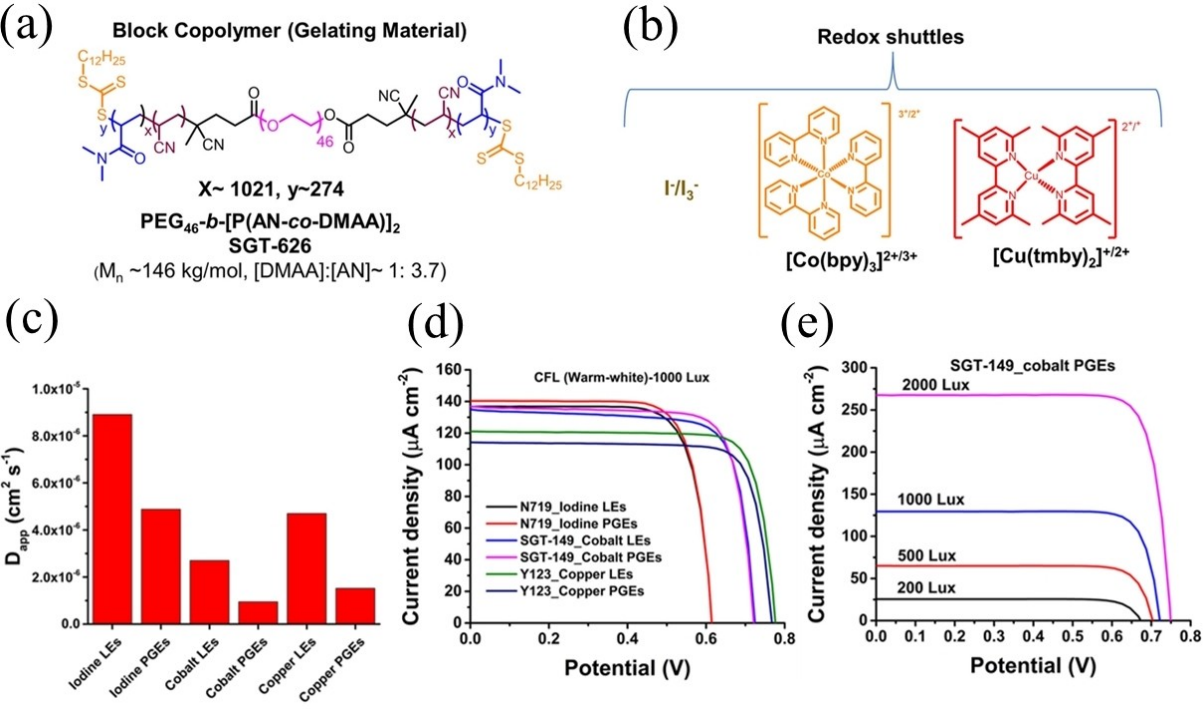
(a) Structure of ABA triblock copolymer as gelating materials, (b) iodide, cobalt and copper based redox shuttles used in this study, (c) Summary of diffusion co‐efficient of iodine, cobalt and copper mediated LEs and polymer gel electrolytes (PGEs), (d) J–V curves of various redox shuttle mediated LEs and PGEs devices under CFL light illumination (1000 lux), and (e) CFL light intensity dependent J–V curve of ss‐DSSC with SGT‐149‐cobalt PGEs. Reproduced from Ref. [195] Copyright (2017), with permission from Elsevier.

In the same year, Hwan Kyu Kim et al. also demonstrated a usage of Co and Cu complex mediated polymer gel electrolyte in DSSC that works efficiently under indoor light condition (CFL;1000 lux) and presented a remarkable PCE of 27.5 and 25 %, respectively.^[195]^ Here, the ABA triblock copolymer is used as gelating material in quasi solid state electrolyte along with the iodide, [Co(bpy)_3_] ^2+/3+^, and [Cu(tmby)_2_]^+/+2^ redox shuttles shown in Figure 26a–b. Though, the diffusion co‐efficient is low for high polymer concentrated electrolyte than LE (Figure 26c), its J_sc_ and photovoltaic performance doesn't affect significantly due to less demand of diffusion of redox shuttle to operate the device under ambient light conditions. Figure 26d represents the J–V curve of various LEs and PGEs based DSSC under low‐intensity conditions (CFL‐1000 lux) which reveals a high J_sc_ for PGEs than its respective liquid electrolytes. The intensity dependent J–V curve of device (Figure 26e) with SGT‐149‐cobalt PGEs showed an enhancement in its performance from 23 to 30 % while increasing the light intensity from 200–2000 lux.

**Figure 27:**
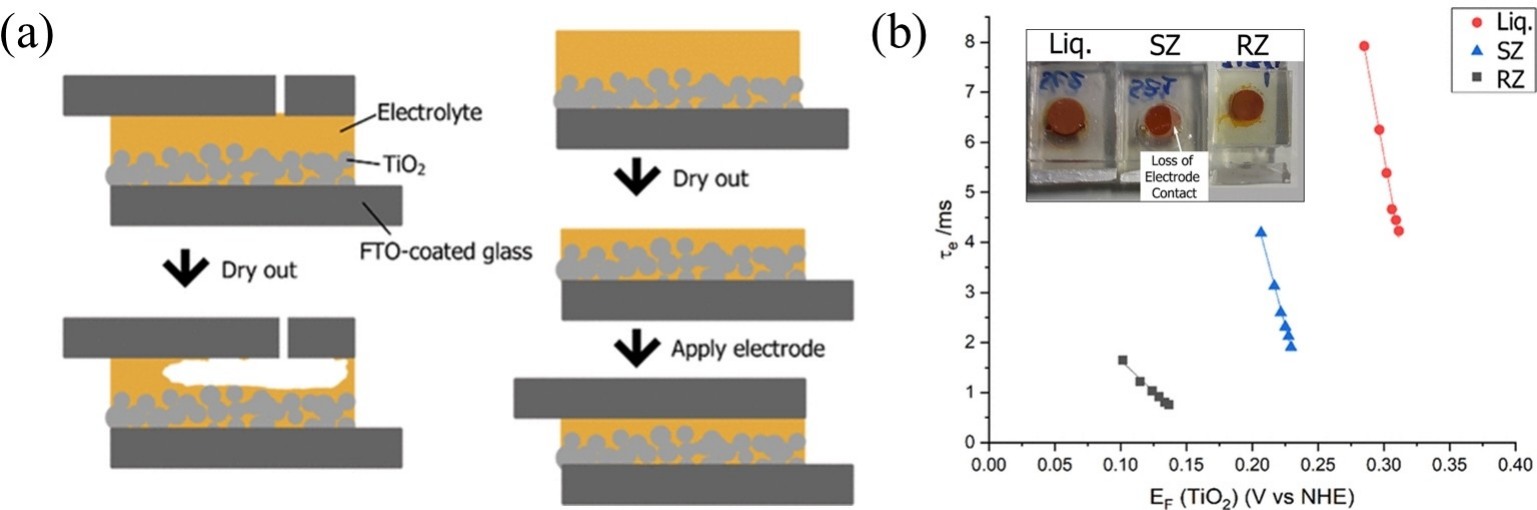
(a) Comparison between well‐established drying process of slow‐zombie (SZ) cell and drying process of a rapid‐zombie (RZ) cell, and (b) electron lifetime studies of liquid, SZ and RZ cells (inset shows photographic image of liquid, SZ, and RZ cells on counter electrode side). Reproduced from Ref. [46] Copyright (2022), with permission from American Chemical Society.

**Figure 28:**
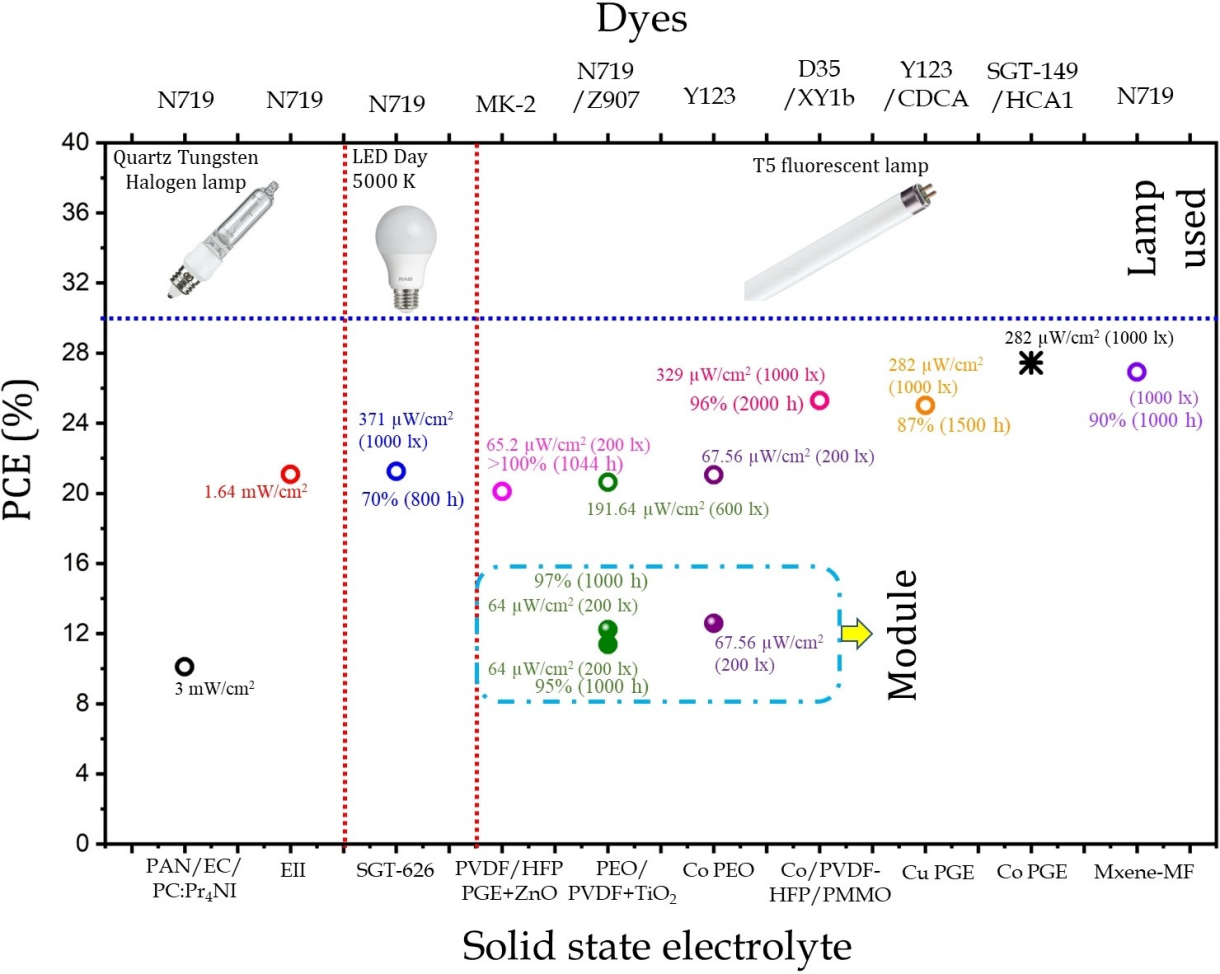
Comparison chart of PCE versus solid‐state electrolyte under different indoor illuminations.

Recently in 2022, Marina Freitag et. al. reported the highest PCE of 21.5 % with LEG4 based zombie cells under the illumination of (5000 K; 1000 lux). This is a high performing device reported for complete solid state DSSC.^[46]^ The variation in the proposed rapid‐zombie (RZ) cells compared with slow‐zombie (SZ) cell (Figure 27a) are desired to enable the volume changes in the cell after drying. In the RZ cells, the volume change happens before the sealing and thus the contact is maintained. Moreover, the transient photovoltage analysis exhibits a slow recombination lifetime (τ_e_) for RZ cells compared with SZ and liquid cells (Figure 27b). The low τ_e_ in RZ cells should possess a higher recombination possibility than other systems. The photovoltaic parameters of ss‐DSSCs and sub‐modules under indoor low‐intensity light conditions are displayed in Figure 28 and Table 4.

**Table 4:** Photovoltaic performance of ss‐DSPV under low light conditions.

Low Intensity (lux)	Solid state polymer electrolyte	Dye	J_sc_ (μA/cm^2^)	V_oc_ (V)	FF (%)	PCE (%)	PCE for LEs (%)	Δη (%)	Stability	Ref.
3 mW/cm^2^	PAN/EC/PC: Pr_4_NI	N719	600	0.61	80	10.1	–	–	–	[186]
1.64 mW/cm^2^	3‐ethyl imidazolium iodide (ETRC)	N719	899	0.636	65.3	21.08	15.5	28	–	[187]
1000 lux	SGT‐626	N719	151.43	0.621	76.63	21.26	19.94	6.6	–	[188]
200 lux 0.0652 μW/cm^2^	PVDF‐HFP PGE + 4 wt % ZnO	MK‐2	24.79	0.669	79.1	20.11	18.91	6.3	>100%‐1044 h	[176]
600 lux	Polyethylene oxide (PEO)/polyvinylidene fluoride (PVDF) with TiO_2_	N719	92.3	0.59	72.5	20.63	–	–	–	[189]
200 lux 67.56 μW/cm^2^	Cobalt polyethylene oxide‐based printable electrolytes	Y123	25.1	0.727	77.9	21.06	16.32	29	–	[190]
1000 lux 329 μW/cm^2^	Co complex‐PVDF‐HFP/PMMA	D35/XY1b	137.6	0.77	78.6	25.3	–	–	96%‐2000 h	[191]
1000 lux (282 μW/cm^2^)	Copper PGE	Y123+CDCA	114	0.768	80.61	25.01	27.06	‐7.5	87%‐1500 h	[195]
1000 lux (282 μW/cm^2^)	Cobalt PGE	SGT‐149+HCA1	137	0.722	78.29	27.45	26.6	3.2	–	[195]
1000 lux	MXene‐Melamine Formaldehyde	N719	196	0.579	71.9	26.92	23.35	15.3	90%‐1000 h	[194]
1000 lux (250.8 μW/cm^2^)	Slow Zombie cell with poly iodide	LEG4	125.2	0.55	0.78	21.5	–	–	–	[41]
200 lux (Module Area: 11.2 cm^2^)	Polyethylene oxide (PEO)/polyvinylidene fluoride (PVDF) with TiO_2_	N719	18.64	0.511	0.765	11.38	–	–	95%‐1000 h	[189]
200 lux (Module Area: 11.21 cm^2^)	Polyethylene oxide (PEO)/polyvinylidene fluoride (PVDF) with TiO_2_	Z907	18.05	0.587	73.9	12.23	–	–	97%‐1000 h	[189]
200 lux 67.56 μW/cm^2^ (Module Area: 11.21 cm^2^)	Cobalt polyethylene oxide‐based printable electrolytes	Y123	16.14	0.671	74.5	12.58	–	–	–	[190]

## 9 Aqueous Quasi Solid‐State Dye‐Sensitized Solar Cells (aq‐qss‐DSSCs)

The results summarized from ss‐DSSC under ambient light conditions highlight the use of DSSC very attractive to power the indoor and low‐power electronics. Nevertheless, the conventional DSSC with organic solvents presents certain limitations such as low boiling points and harmful environmental effects. This has prompted research into aqueous‐based electrolyte systems, known for their cost‐effectiveness, abundant water resources, and improved environmental compatibility.[^197^, ^198^, ^199^, ^200^, ^201^] The framework of quasi‐solid‐state or solid‐state electrolytes demonstrates relative stability, providing an additional opportunity to customize the light propagation in DSSC research. Zhongze Gu et al.^[202]^ achieved an enhanced PCE of 1.8 % which is ca. 22 % higher than conventional quasi‐gel electrolyte without any water in it. In this study, hydrogels featuring an inverse opal structure (IOS) were synthesized using various SiO_2_ opal films. These hydrogels were then employed as a host material to create fully water‐based gel electrolytes with a photonic band gap (PBG). This innovative approach enhances DSSC's light harvesting capabilities through the phenomena of back reflection and photon scattering, while also reducing the Warburg impedance associated with I^−^/I_3_^−^ diffusion.

Several studies have explored the impact of water‐based electrolyte systems in ss‐DSSC under 1‐sun conditions. However, there is limited existing literature on *aqueous‐q*ss‐DSSC systems functioning effectively under ambient low‐intensity light conditions. In another study conducted by Tarek H. Ghaddar et al.^[203]^ a water soluble polypyridyl copper complex, [Cu(I)(dc‐dmbpy)_2_Cl/Cu(II)(dc‐dmbpy)_2_Cl_2_, dc‐dmbpy=6,6′‐dimethyl‐2,2′‐bipyridine‐4,4′‐dicarboxylate] with C106 Dye‐sensitized TiO_2_ was utilized in ss‐DSSC and realized the device PCE of 7.5 % under indoor light condition (100 lux; 24.2 μW ⋅ cm^−2^).

## 10 Summary and Outlook

In recent years, researchers have extensively focused on the usage of solid‐state materials in Dye‐sensitized solar cells (ss‐DSSCs) to increase their stability, where the liquid electrolyte is replaced with the polymer and gel type electrolyte materials. However, the performance of ss‐DSSC is lower than conventional DSSCs due to low physical contact between the sensitizer and the solid electrolyte. To realize high photovoltaic performances in ss‐DSSCs, a variety of photosensitizers were developed in recent days. In this review, we summarize the advances on photosensitizers for ss‐DSSCs and their photovoltaic performance under 1‐sun and ambient low‐intensity light conditions. Photosensitizers are the key component which plays a vital role in ss‐DSSC for both light harvesting (converting the incident light photon into electricity) and charge injection (through the anchoring unit). Herein, we classified them into three categories based on the chemical structure including Ru‐complexes, Zn‐porphyrin, and metal‐free organic sensitizers.

Ru‐polypyridyl complexes were the first‐generation sensitizers investigated for the DSSC research. Extensive focus on these complexes has provided a useful pathway for the development of other types of sensitizers. Molecular engineering strategies of Ru‐based sensitizers have been employed to enhance the light harvesting capability and long‐term stability. The use of thiocyanate ligands typically leads to red‐shifts in optical absorption, while the ancillary ligands with extended π‐conjugation increases the molar extinction coefficient (ϵ). After careful optimization, DSSC with Ru‐photosensitizers have achieved a PCE of more than 10 %. Recently, a record PCE of 13 % was reported using zinc porphyrin sensitizers in combination with the organic dye as co‐sensitizers and cobalt‐based redox mediators. Zn‐porphyrin dyes could exhibit an impressive absorption profile from visible to near‐infrared (NIR) regions with high molar extinction coefficients.

The introduction of donor and acceptor units to the periphery of the porphyrin macrocycle unit was one of the most important strategies in the design of high‐performance porphyrin dyes. Through this molecular design, a push‐pull structure is realized that promotes a wide absorption in visible region and a directional electron transfer from the donor to the anchoring acceptor unit. Metal‐free organic dyes offer several advantages over Ru‐complexes and porphyrin‐based dyes. The simplest and most well‐known synthetic procedures allow the easy tuning of the optical properties and are more compatible with a low‐cost, and large‐scale production. Metal‐free organic dyes are highly suitable for the applications in ss‐DSSC due to their high ϵ value. The typical structure of this class of dyes is based on donor‐spacer‐acceptor (D–π–A) configuration, where the changes in this configuration are highly influencing the photovoltaic performance. Triphenyl amine and indoline derivatives are the most studied electron donors and the cyanoacrylic unit is the commonly used electron acceptor in metal free dyes. Several π‐spacer units have been studied, containing auxiliary acceptor groups or rigidified aromatic units to increase their planarity.

Typically, the performance of monolithic ss‐DSSCs suffer, due to its difficulty in thickness control, because of less infiltration in HTMs. Hence, co‐sensitization of suitable new and complementary dyes could lead to higher PCEs than other conventional DSSCs. Also, careful energy alignment matching between the co‐sensitizers and HTMs will be required for going forward. The alternative device architectures (like zombie cells), which eliminate the problems of less HTM infiltration and low TiO_2_ thickness should be explored, which would result in high device performance towards the proliferation of the ss‐DSSC technology in the photovoltaics market. The immobile nature of the device components and its related fast electron transport processes require direct regeneration of the oxidized dye, which can be achieved by exploring a weak connection between the dyes and HTMs.

This could significantly improve the performance of ss‐DSSCs due to the physical contact and the barrier for energy loss is eliminated between the sensitizer and HTM. Under the artificial light (ambient low‐intensity light) conditions, the ss‐DSPV has gained significant interest because of its potential as a practical and sustainable energy source for low‐power based internet of things (IoT) applications. When a transparent quasi or all solid‐state electrolyte is employed, the resulting ss‐DSPV can be even transparent in visible region which could be more useful for bifacial applications such as in BIPV that can be directly integrated with portable electronic devices like wireless sensor node (WSN) and IoTs. In this review, the recent developments in ss‐DSPV under artificial light illumination conditions were thoroughly examined with respect to variety of photosensitizers utilized in ss‐DSPVs. The spectral match of photosensitizers with artificial light sources, suitable energy levels and compact molecular packing at the TiO_2_ surface can reduce the charge recombination and improve the overall PV performance. Furthermore, to improve the device performance under ambient light conditions, the down/up‐conversion materials can be utilized for spectral tuning, which can significantly enhance the device efficiency as future prospects.

## Conflict of interest

The authors declare no conflicts of interest.

11

## Biographical Information

*Bommaramoni Yadagiri has been working as an Assistant Professor in the Department of Energy and Materials Engineering, Dongguk University, Seoul, Republic of Korea, since 2022. He was a postdoctoral researcher with the same department, at Dongguk University, Seoul, Republic of Korea, from 2021 to 2022. He obtained his Ph.D. degree in Chemical Science at Polymers and Functional Materials Department, CSIR‐Indian Institute of Chemical Technology (CSIR‐IICT), Hyderabad. His research interests are focused on the design and synthesis of novel photo active organic materials for organic photovoltaics, perovskite solar cells, and Dye‐sensitized solar cells*.

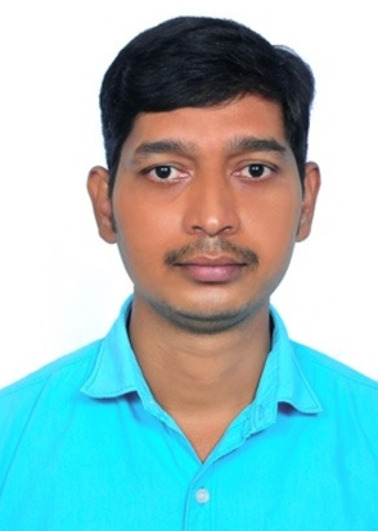

## Biographical Information

*Ashok Kumar Kaliamurthy has been working as an Assistant Professor in the Department of Energy & Materials Engineering at Dongguk University in Seoul, Republic of Korea, since 2023. He was a Korea Research Fellow (KRF) with the same department at Dongguk University (2019‐2023). He was a National Post‐Doctoral Fellow (NPDF) in the Department of Chemistry at Anna University in Chennai, India (2017‐2019). He received his Ph.D. and M.Phil. in Physics at the Department of Nuclear Physics, University of Madras, Tamil Nadu, India. His current research interests are focused on the development of indoor photovoltaics (dye‐sensitized and perovskite solar cells) by utilizing nanophosphor, and flexible supercapacitors*.

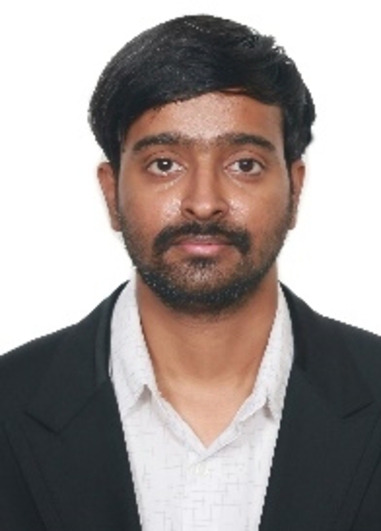

## Biographical Information

*Kicheon Yoo has been a research professor with the Department of Energy & Materials Engineering, Dongguk University, Seoul, Republic of Korea, since 2018. He was a postdoctoral researcher with the Department of Energy & Materials Engineering, Dongguk University, Seoul, Republic of Korea, from 2016 to 2018. He received his Ph.D. degree from Yonsei University, Republic of Korea, in 2016. His current research concentrates on the fundamental issues in the synthesis and characterization of nanomaterials for photoelectrochemical cells (Dye‐sensitized solar cell, flexible Dye‐sensitized solar cell, and perovskite solar cell) and developments of photo‐electrochemistry/interfacial nanoengineering‐based electrode*.

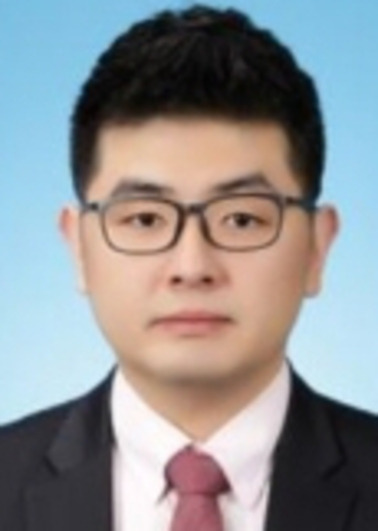

## Biographical Information

*Hyeong Cheol Kang received his B.S. from Dongguk University (2019) in Korea. He is currently an MS PhD integrated student studying electrochemistry under the supervisor of Prof. Jae‐Joon Lee in the Department of Energy & Materials Engineering at Dongguk University. His current research is focused on developing advanced nanomaterials for Photoelectrochemical cells and their applications*.

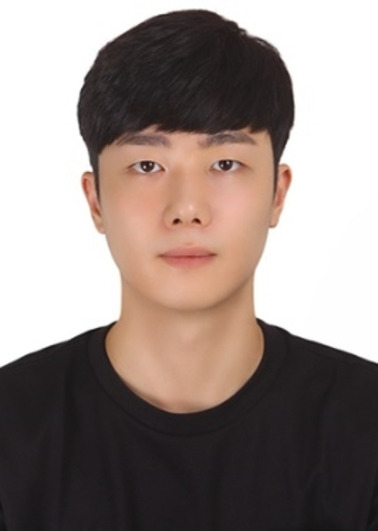

## Biographical Information

*Junyeong Ryu received his B.S in the Department of Energy and Materials Engineering. He is a Ph.D. student, proceeding with a degree in Lab of Nanochemistry and Photoelectrochemistry, Department of Energy & Materials Engineering, Dongguk University, Seoul, Republic of Korea. His current research is focused on electrochemistry and main research background is dye‐sensitized solar cells*.

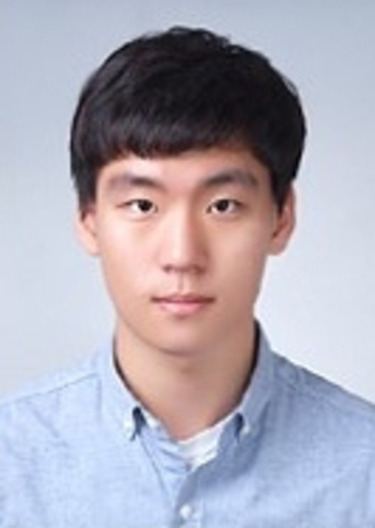

## Biographical Information

*Francis Kwaku Asiam obtained his B.Sc. degree in chemistry from Kwame Nkrumah University of Science and Technology (KNUST), Ghana, in 2017, and his master's degree in engineering from Dongguk University, Korea, in 2022. He is currently a Ph.D. student under the supervision of Prof. Jae‐Joon Lee in the Department of Energy & Materials Engineering, Dongguk University, Korea. His research focus is on developing materials for solar energy (dye‐sensitized solar cells) applications and their related fundamental kinetic and thermodynamic properties*.

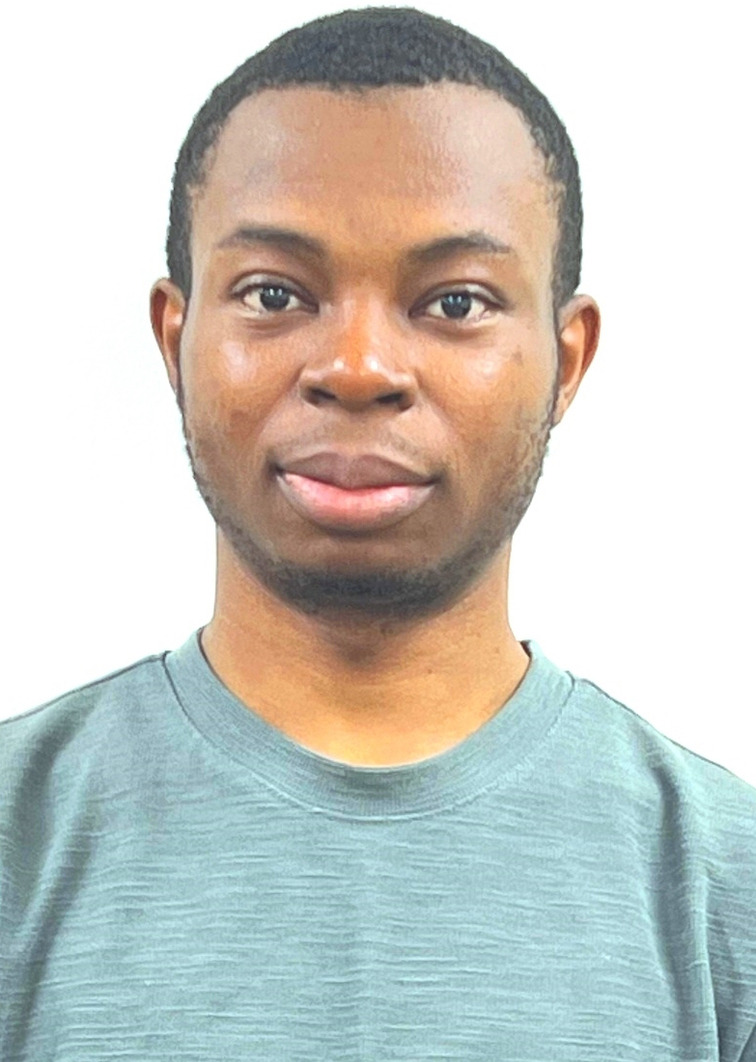

## Biographical Information

*Jae‐Joon Lee has been a Professor with the Department of Energy & Materials Engineering, Dongguk University, Republic of Korea, since 2016. He was a professor with the Department of Applied Life Science, Konkuk University, Korea, from 2004 to 2015. He received his M.S. in chemistry from Seoul National University. He received his Ph.D. in chemistry from Case Western Reserve University and worked as a postdoctoral scholar with the California Institute of Technology, California. His research interests include dye‐sensitized solar cells and perovskite solar cells as well as the development of electrochemical energy conversion systems and biosensors based on surface modification of electrodes*.

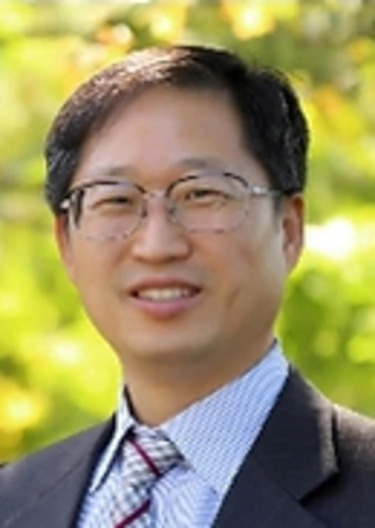

## Data Availability

Data sharing is not applicable to this article as no new data were created or analyzed in this study.
